# Federated generative prompt learning with vision foundation models: universal efficient multi-center medical image analysis

**DOI:** 10.1038/s41746-026-02866-1

**Published:** 2026-06-10

**Authors:** Xi Lin, Yuliang Chen, Jun Wu, Chang Xu, Jianhua Li, Xiu Su

**Affiliations:** 1 School of Computer Science, Shanghai Jiao Tong University, Shanghai, China; 2 Shanghai Key Laboratory of Integrated Administration Technologies for Information Security, Shanghai, China; 3 School of Computer Science, Faculty of Engineering, The University of Sydney, Sydney, Australia; 4 Big Data Institute, Central South University, Hunan, China; 5 National Engineering Research Center for Medical Big Data Application Technology, Changsha, China

**Keywords:** Computational biology and bioinformatics, Engineering, Health care, Mathematics and computing, Medical research

## Abstract

Federated medical AI revolutionizes multi-center collaboration, while communication cost, data scarcity, and heterogeneity still limit its practical deployment. Foundation models (FMs) offer a promising avenue for addressing these challenges, owing to their generalization capabilities and efficient adaptability to medical tasks. Here, we present Federated Generative Prompt Learning (Fed-GPL), a universal and efficient framework for multi-center medical image analysis. It collaboratively trains a prompt generator that produces customized prompts for each patient, capturing patient-specific variations and enabling precise medical diagnosis. Fed-GPL is compatible with various vision FMs and medical tasks, such as Vision Transformer (ViT) for diabetic retinopathy and melanoma classification, and Segment Anything (SAM) for polyp and prostate segmentation. Fed-GPL outperforms traditional models and full fine-tuning methods, with only 8.26% and 6.55% of the total FM parameters being trained across classification and segmentation tasks, while converging within just 15 communication rounds. For low-resource settings, Fed-GPL maintains its performance with 5% of the original training data.

## Introduction

The rapid development of digital technology in healthcare has led to the accumulation of vast amounts of patient data in hospitals and medical institutions, including electronic medical records, medical images, and genomic data. The scale and complexity of medical data exceed the capabilities of traditional data analysis methods. Artificial intelligence (AI) technologies, particularly deep learning and machine learning^1^, have demonstrated strong potential to extract valuable patterns and insights from these datasets, creating unprecedented opportunities for precision medicine^2,3^ and intelligent diagnosis^4,5^. In medical image analysis, AI can automatically analyze imaging modalities such as dermoscopy, endoscopy, and MRI to identify malignant pathological regions, including breast cancer^6–8^, lung nodules^9,10^, liver tumors^11^, thyroid nodules^12^, and brain tumors^13–15^, significantly improving the efficiency and accuracy of medical diagnoses. The accuracy of medical AI rivals, and in some cases surpasses, that of human experts, especially in detecting early-stage lesions that are challenging to identify, where AI demonstrates heightened sensitivity.

Medical AI models are typically trained in a centralized manner, which relies on collecting medical data with highly sensitive personal information, including patient medical histories, genomic data, diagnostic records, and medication usage. Data regulations such as the Health Insurance Portability and Accountability Act (HIPAA) in the United States^16,17^ and the General Data Protection Regulation (GDPR) in the European Union^18,19^ mandate stringent privacy protections for sensitive health data. It is crucial to enable collaborative AI model training in multi-center scenarios while ensuring patients’ privacy. Federated learning (FL) has emerged as a promising solution^20,21^, enabling hospitals to collaboratively train models without directly sharing sensitive data, thereby preserving patient privacy. Federated training of medical AI models among institutions^22–25^ is especially valuable in healthcare, where strict data regulations tightly control data access.

However, federated medical AI still faces several significant challenges. First, as the scale of model parameters increases, the frequent exchange of model parameters in FL leads to higher bandwidth consumption and latency, hindering the efficiency of collaborative model training. Second, obtaining data on rare diseases or specific patient groups is challenging. The scarcity of data on local devices^26^ can result in overfitting of local models, thereby reducing their generalization capability. Moreover, variations in multi-center medical image analysis, due to differences in medical equipment, image acquisition conditions, and operational practices, introduce feature shifts^27^. These shifts can compromise the generalization capability of medical models and limit their adaptability across hospitals.

The emergence of large-scale foundation models (FMs) has provided new opportunities to address long-standing challenges in federated medical image analysis. Representative FMs include GPT (Generative Pre-trained Transformer)^28,29^, ViT (Vision Transformer)^30,31^, and SAM (Segment Anything)^32^, which have demonstrated strong transferability across natural language processing, computer vision, and multimodal learning tasks. Owing to their large-scale pretraining, FMs can be adapted to downstream medical tasks with limited labeled data, effectively reducing the reliance on extensive annotation and alleviating data scarcity in medical AI. Integrating medical AI with FMs^33,34^ enables the transfer of robust feature representations and generalizable pattern recognition capabilities to downstream medical image analysis. To efficiently adapt FMs in resource-constrained federated settings, parameter-efficient fine-tuning (PEFT) methods^35–38^ have emerged as a practical solution. By freezing the majority of backbone parameters and only updating lightweight modules, PEFT substantially reduces the number of trainable parameters, thereby lowering communication costs during federated optimization. Among various PEFT strategies, prompt learning^36,39^ is particularly attractive due to its flexibility and efficiency.

However, existing attempts to incorporate prompt learning into federated learning^40,41^ typically rely on naive parameter averaging across hospitals, which fails to adequately address severe data heterogeneity arising from differences in imaging devices, acquisition protocols, and patient populations. In practice, such approaches usually aggregate trainable prompt parameters across clients and reuse a single shared prompt at inference time, implicitly assuming that a globally averaged representation can generalize well across heterogeneous medical centers. This assumption is often violated in real-world clinical settings, where variations across institutions and even within individual hospitals introduce substantial distribution shifts. To address this limitation, a key insight is that federated collaboration should emphasize sharing transferable task knowledge, rather than directly enforcing uniform prompt representations during inference. From this perspective, global aggregation is used to capture common diagnostic patterns across institutions, while the actual model adaptation should remain flexible to accommodate sample-level variations. Such a design decouples global knowledge sharing from local adaptation and offers a principled alternative to naive parameter averaging when dealing with heterogeneous medical data. In this work, we focus on the intertwined challenges of data scarcity, communication bottlenecks, and cross-center heterogeneity in multi-center medical image analysis, and aim to develop a universal and efficient federated prompt learning framework that enables robust adaptability across diverse clinical environments.

**Fig. 1:**
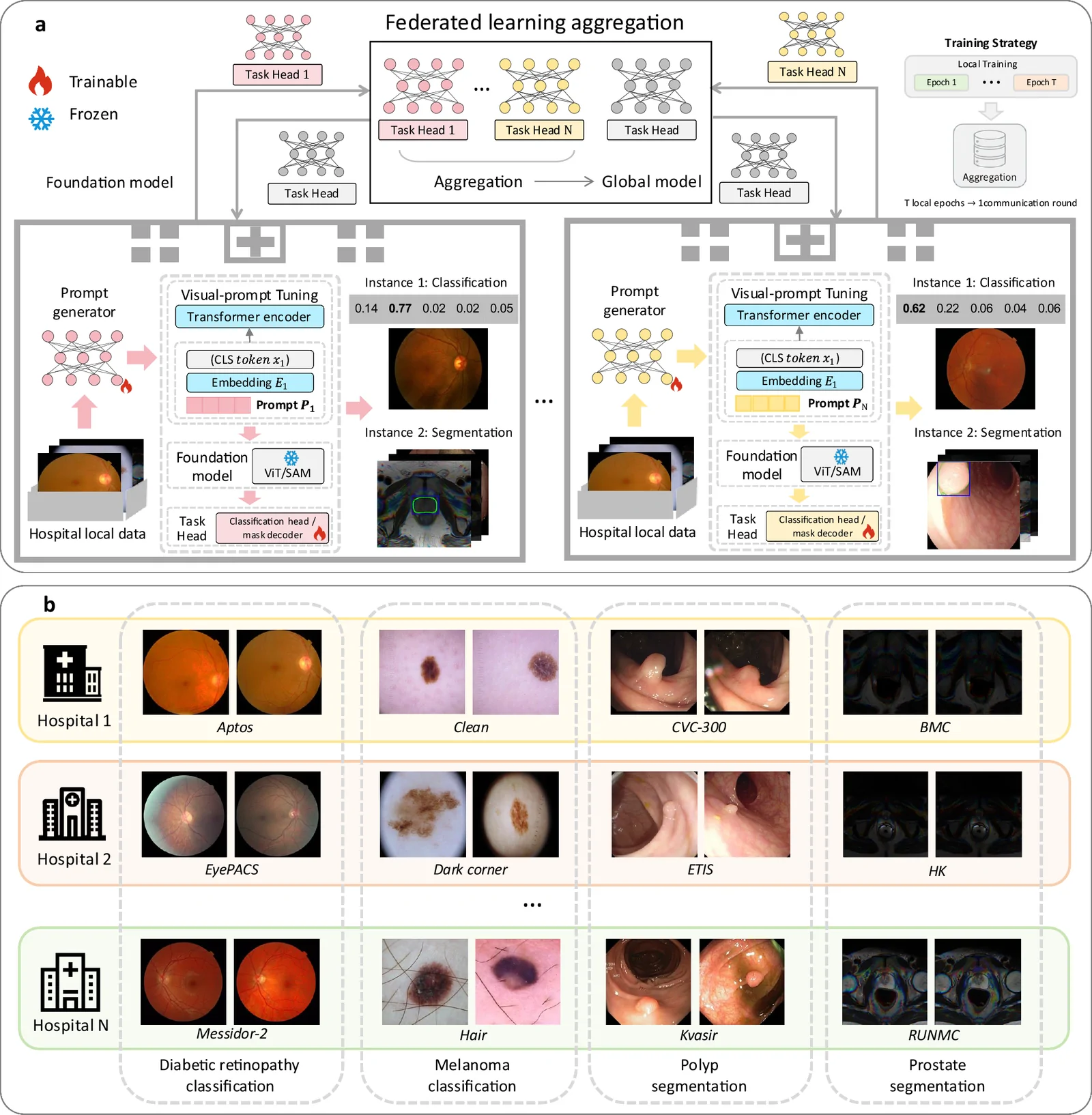
Overview of the Fed-GPL framework. **a** The framework consists of a central server and several distributed hospitals. A downstream task-related vision foundation model (ViT or SAM) is deployed at local hospitals and fine-tuned with visual prompts. The local hospitals and the central server collaboratively train the prompt generator and prediction module. Fed-GPL supports both medical classification and segmentation tasks. **b** We consider a multi-center scenario, where data from different hospitals exhibit diverse styles and domain characteristics. We selected two distinct datasets for each task. Notably, the melanoma classification task is partitioned by feature characteristics rather than by data source.

**Fig. 2:**
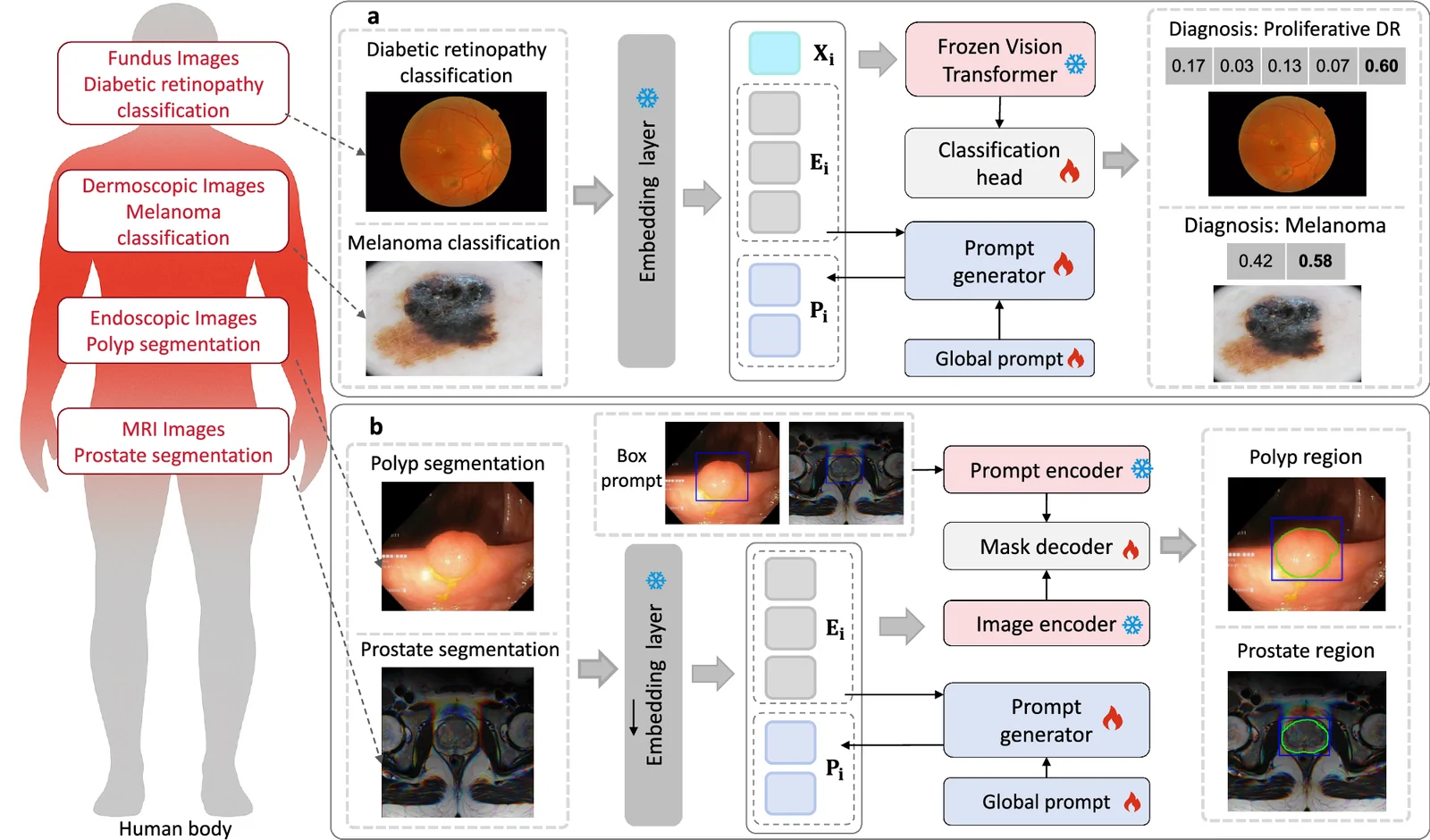
Inference process of Fed-GPL. **a** The prompt generator integrates the patch embedding of the input image with the global prompt to generate a personalized prompt, which is concatenated with the image embedding for feature extraction. The output token is then fed into the classification head for prediction. **b** In the segmentation task, visual prompts are generated in a similar manner and concatenated with the input of the image encoder. The mask decoder combines the image embedding, the bounding box prompt embedding, and the output token to produce the final mask.

We propose a *Federated Generative Prompt Learning* (Fed-GPL) framework for fine-tuning large-scale vision foundation models in multi-center scenarios. Figure 1a illustrates the training process of our framework, where a foundation model with frozen backbone parameters is deployed at each local hospital to facilitate training on low-resource medical data. In Fed-GPL, participating hospitals collaboratively train an instance-specific prompt generator and relevant prediction modules, enabling communication-efficient global aggregation. The prompt generator leverages the cross-attention mechanism^42^ to integrate task knowledge from a global prompt with personalized information from individual patch embeddings, generating customized prompts for each medical sample. By dynamically adapting prompts to the unique characteristics of each patient case, this module addresses two long-standing challenges in medical federated learning. First, it mitigates the severe inter-clients data heterogeneity caused by differences in imaging devices, acquisition protocols, and patient demographics, which often hinders the transferability of shared models. Second, it bridges the semantic gap between global medical knowledge and local patient-specific features, enabling the model to generalize well across institutions while maintaining sensitivity to subtle pathological variations. This design allows the prompt generator to act as a flexible knowledge interface, capturing globally consistent diagnostic patterns while tailoring the decision-making process to each sample, thereby improving both cross-site robustness and instance-level precision without increasing communication or privacy risks. Figure 2 shows the corresponding inference pipeline. We evaluate Fed-GPL in medical image classification and segmentation tasks, each utilizing two cross-hospital datasets. Extensive experimental results validate that Fed-GPL effectively addresses domain shift across hospitals, improving classification and segmentation performance compared to baseline methods in low-resource conditions. Specifically, Fed-GPL reduces communication costs and enhances resistance to privacy attacks relative to traditional FL methods. We anticipate that our proposed method could become a valuable paradigm for handling diverse medical modalities in multi-institutional medical image analysis, particularly in low-resource settings.

## Results

### Multi-center medical datasets

As illustrated in Fig. 1b, we consider a multi-center scenario where hospital *i* possesses data exclusively from domain *i*, each with a distinct image style including different shooting devices, different lighting conditions, or different impurities in medical images. Our paper focuses on medical classification and segmentation. For each task, we utilize two cross-hospital datasets: medical image classification is performed on diabetic retinopathy and melanoma datasets, while medical image segmentation involves prostate segmentation and polyp segmentation.

#### Diabetic retinopathy classification

Diabetic retinopathy (DR)^43,44^ is an eye complication caused by diabetes. Damage to the retinal blood vessels due to high blood sugar can lead to vascular leakage and retinal detachment, among other complications. The DR dataset is a collection of fundus image datasets used for lesion classification, with categories including No DR, Mild, Moderate, Severe, and Proliferative DR. We select four DR datasets to form a cross-domain dataset, which includes: *EyePACS* (35,126 images), *Messidor-1* (1200 images), *Messidor-2* (1744 images), and *APTOS 2019* (3662 images). EyePACS and APTOS 2019 follow a five-grade DR severity labeling scheme ranging from 0 (no DR) to 4 (proliferative DR), which is consistent with widely adopted clinical grading protocols. These labels are provided through clinician grading processes as part of large-scale public challenges. Messidor-1 contains 1200 fundus images captured with a 45-degree field-of-view using non-mydriatic cameras, and includes DR grading annotations commonly used for benchmarking DR detection methods. Messidor-2 is composed of a larger set of fundus examinations, typically including two macula-centered images per examination, and is frequently used to assess DR-related classification performance, although a single unified ground-truth labeling standard is not consistently released across all public versions.

#### Melanoma classification

Melanoma is a type of skin cancer^45^ that occurs when melanocytes, the cells responsible for producing skin pigment, become cancerous. The ISIC2019 dataset^46^ consists of 25,331 dermoscopic images collected from multiple clinical institutions using dermatoscopes under standardized imaging conditions. The dataset provides diagnostic annotations derived from histopathological examination, expert consensus, or clinical follow-up, and is commonly regarded as a reliable benchmark for melanoma classification. In addition to image-level labels, ISIC 2019 includes auxiliary metadata for many samples, such as patient age, sex, and anatomical site of the lesion, although these demographic attributes are not consistently available for all images. We focus on the diagnosis of melanoma, only considering two classes: malignant (melanoma) and benign. Based on the annotated artifacts^47^, we divide the ISIC2019 dataset into nine domains and select five domains for the experiments: *clean* (4732 images), *dark corner* (4609 images), *gel bubble* (3550 images), *ruler* (1158 images), and *hair* (8970 images).

#### Polyp segmentation

Polyp segmentation is a fundamental task in medical image analysis, playing a crucial role in the diagnosis and treatment of colorectal cancer. The polyp dataset typically involves endoscopic images of the gastrointestinal tract, especially colonoscopy images, for the detection and segmentation of polyps. We collect endoscopic images from five publicly available datasets, each from different institutions or with different data types. *Kvasir*: Collected by Vestre Viken Health Trust in Norway, with 1000 polyp images of the gastrointestinal tract. *CVC-ClinicDB*: Created by the Computer Vision Center (Centro de Visión por Computadora, CVC) in Barcelona, Spain, with 612 images collected from 29 colonoscopy video sequences. *CVC-ColonDB*: A static image dataset created by CVC, with 380 images. *CVC-300*: Created by CVC, including 60 polyp images. *ETIS*: Created in collaboration between the Mines ParisTech and the Laboratory for Research in Imaging and Biomechanics (LARIB) in France, with 196 polyp images. The annotation standard for all polyp datasets is pixel-wise binary segmentation, where each image is paired with a ground-truth mask delineating polyp regions. In Kvasir-SEG, the segmentation masks are manually annotated by medical experts and subsequently verified by experienced gastroenterologists. The CVC datasets similarly provide expert-annotated binary masks and are commonly used as reference benchmarks in polyp segmentation studies. ETIS-Larib is known to be particularly challenging due to large variations in polyp size, shape, and appearance.

#### Prostate segmentation

Prostate segmentation in magnetic resonance imaging (MRI) is clinically important as it helps determine prostate volume ^48^, which aids in evaluating prostate diseases, predicting the pathological stage of prostate cancer, and assessing treatment response. The prostate segmentation dataset^49^ consists of preprocessed T2-weighted prostate MRI images and ground truth masks. The dataset is organized into multiple sites, each corresponding to a different institution or acquisition protocol. It is collected from six different medical institutions: *RUNMC* (30 cases, 387 images), *BMC* (30 cases, 354 images), *HCRUDB* (19 cases, 449 images), *UCL* (13 cases, 162 images), *BIDMC* (12 cases, 289 images), and *HK* (12 cases, 146 images). Site-level information includes the number of cases, scanner field strength, in-plane and through-plane resolution, scanner manufacturer, and whether an endorectal coil was used. This explicit multi-center decomposition allows the evaluation of domain shift caused by heterogeneous imaging protocols.

### Data preprocessing

Different foundation models require different input image sizes. The ViT-base and SAM-base models require input images of size 3 × 224 × 224 and 3 × 1024 × 1024, respectively. For ViT-based models used in classification tasks (DR and melanoma), we apply a unified data augmentation and preprocessing pipeline during training, including random horizontal and vertical flipping, random resized cropping to 224 × 224 with a scale range of [0.75, 1.0], random rotation within 45^∘^, and color jittering on the hue channel. All images are then converted to tensors and normalized using ImageNet statistics. This pipeline is consistently applied across all domains to harmonize data distributions while preserving domain-specific visual characteristics. For the SAM ViT-base model, we utilize the ResizeLongestSide method to resize the original image into the required input resolution, without introducing additional data augmentation, following the standard SAM preprocessing protocol. Specifically, for the compared segmentation methods, including U-Net and DeepLabV3+, the size of the input image needs to be a multiple of 32. To this end, we add padding to the images and their corresponding masks so that their sizes are multiples of 32. For polyp segmentation, images are loaded in RGB format, and the corresponding ground-truth masks are converted to binary maps via thresholding. To ensure spatial alignment, identical padding operations are applied to both images and masks. Furthermore, the padding sizes of all data in the same batch are required to be identical, while the sizes of polyp segmentation images vary unevenly. To address this problem, we cluster original images by image size and create corresponding subsets. The largest subset was then chosen as the target for the subsequent dataset partition.

Another crucial aspect of data processing is dataset partitioning. We divide the datasets into training and test sets. In cross-domain medical datasets, the number of samples varies across different domains. To ensure a fair comparison, we allocate the same number of training and test samples to all clients. Specifically, for the classification tasks, we set the number of testing samples to 200 and the number of training samples to 800. For the segmentation task, we selected 20 samples as the test set and 40 samples as the training set. The data quantities were determined by the smallest domain in the dataset. In future experiments, we can further reduce the training data through random selection. In our partition strategy, we perform five independent splits, ensuring variability in each partition to reflect different domain characteristics. We do not fix the class distribution across splits to increase the robustness of the experiments. Throughout the data partition process, the original class label distribution within each domain is preserved to maintain inherent label heterogeneity and avoid introducing artificial class imbalance.

### Experiment setup

We evaluate the performance of our method by comparing it with the current SOTA methods in multi-center scenarios. For the classification task, we benchmark against both ResNet^50^ and Vision Transformer (ViT)^30^ backbones. In the **Fed-ResNet** baseline, a ResNet-50 model is trained from scratch. For the ViT-based comparisons, we employ the ViT-Base model trained from scratch (**Fed-ViT**). Additionally, we consider a fully fine-tuned ViT model (**Fed-ViT-Full**), where all pre-trained parameters are updated during downstream training. Similar to the classification task, we include full fine-tuning on SAM^51^ as a comparison method (**Fed-SAM**). For the segmentation task, we compare our method against state-of-the-art models, including U-Net^52^ and DeepLabV3+^53^. Due to the limited size of local datasets, training segmentation models from scratch is challenging. Therefore, we adopt federated full fine-tuning of ImageNet-pretrained U-Net (**Fed-U-Net**) and DeepLabV3+ (**Fed-DeepLabV3+**) as baseline methods. For the segmentation foundation model, we utilize SAM with the ViT-B backbone (SAM ViT-B), generating predictions based on box prompts defined by the top-left and bottom-right corner points. To ensure a fair and consistent comparison with the SAM-based method, we augment both U-Net and DeepLabV3+ by adding an additional binary box channel to their input layers. This modification aligns the input conditions across all models, ensuring that each model receives similar information for prompt-based segmentation tasks. Specifically, the SAM-based method provides the box prompt, and in our experiments, we adopt the approach from MedSAM^51^, where the bounding box is transformed into a binary mask and used as an additional input channel. This guarantees that the comparison is unbiased and that all models are evaluated under equivalent conditions. Similar to the classification task, we include a fully fine-tuned SAM model as a comparison (**Fed-SAM**)^51^.

### Implementation details

Our experiments were conducted on a standalone machine equipped with an NVIDIA RTX A40 GPU with 48 GB of memory. Our framework was implemented using Python 3.11.6, CUDA 12.2, and PyTorch 2.3.0. Python packages for data analysis and image visualization include torchvision 0.18.0, numpy 1.26.4, matplotlib 3.8.4, pandas 2.1.4, scipy 1.13.0, and opencv 4.3.0. We directly utilized state-of-the-art segmentation models with pre-trained weights from the segmentation_models.pytorch library. All trainable modules are optimized using the AdamW optimizer.

In the hyperparameter configuration, we uniformly set the local training to 2 epochs and the global training to 30 rounds. For classification tasks, the learning rate is set to 5 × 10^−6^, the weight decay to 1 × 10^−4^, the number of local training samples to 400, and the batch size to 32. For segmentation tasks, the learning rate is uniformly set to 1 × 10^−4^, the weight decay to 5 × 10^−4^, the number of local training samples to 30, and the batch size is set to 2. The loss functions used for both classification and segmentation tasks are defined in the Evaluation Metrics section. Specifically, classification models are trained with the cross-entropy loss, while segmentation models are optimized using a combination of Binary Cross-Entropy (BCE) loss and soft Dice loss. Regarding visual prompt configurations, different prompting strategies and prompt lengths are adopted for different downstream tasks to balance adaptability and efficiency. For diabetic retinopathy classification, we selected VPT-Shallow (VPT-S) with a prompt length of 10. For melanoma classification, we chose the VPT-Deep (VPT-D) method with a prompt length of 4. For polyp segmentation, we also opted for VPT-Deep, but with a prompt length of 2. For prostate segmentation, we selected the VPT-Shallow strategy with a prompt length of 1.

### Performance evaluation

**Fig. 3:**
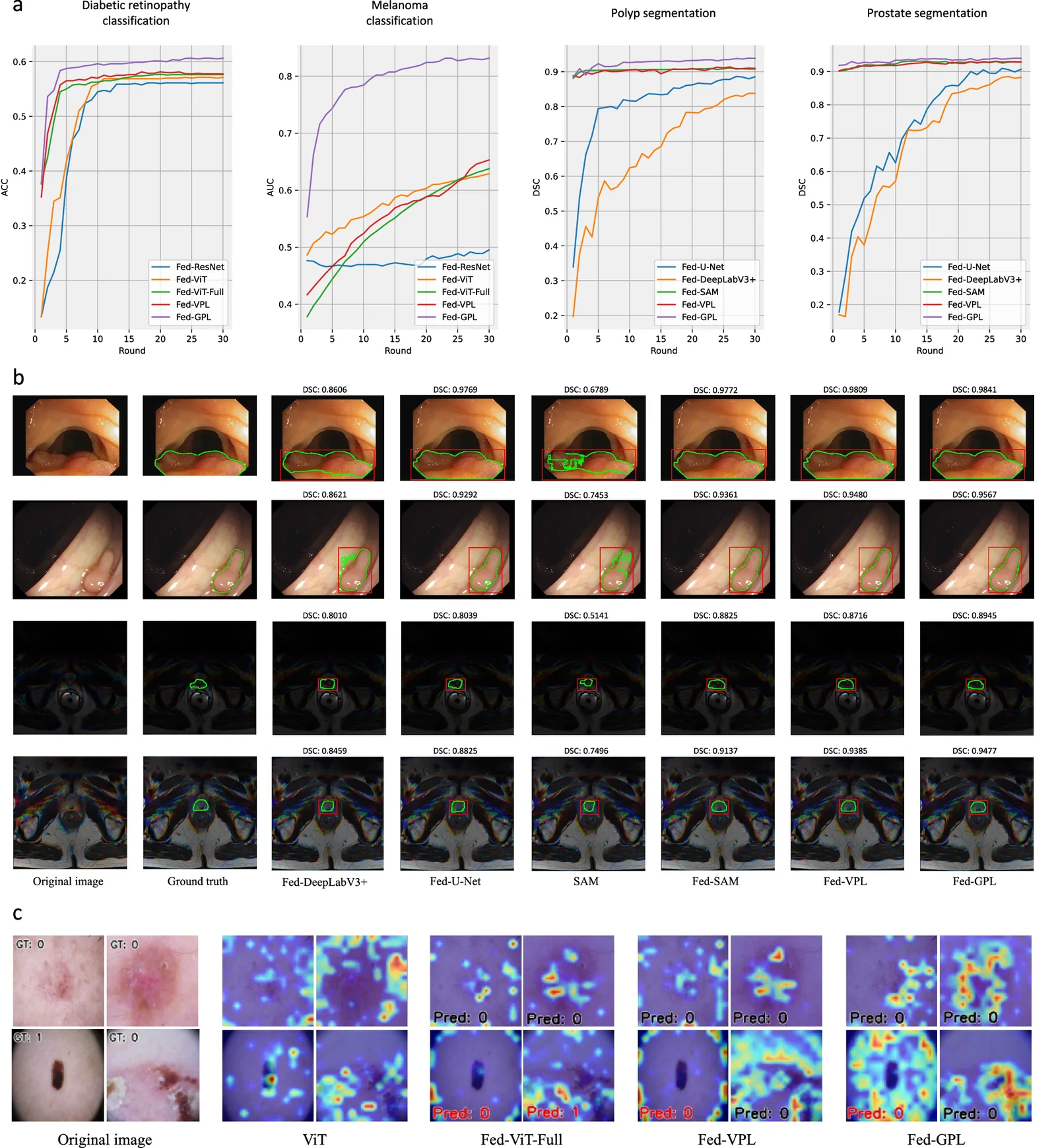
Visualization of comparative experimental results. **a** The convergence curves for the four downstream tasks. **b** Visualization results for the segmentation tasks, including original images, ground truth masks, and segmentation results of different methods. Red boxes denote box prompts, and green contours denote segmentation boundaries. **c** Grad-CAM visualizations for different models. The first column shows the original images and ground truth annotations, and the remaining columns show attention heatmaps. The predicted class (0 or 1) is shown in the lower-left corner.

Table 1 presents a performance comparison for the classification task. In diabetic retinopathy classification, we use accuracy (ACC) to evaluate the performance of the five-class classification problem. The introduction of prompt learning has achieved improvements in model classification performance while significantly reducing resource consumption. Fed-GPL outperforms the comparison methods across all domains. In terms of average accuracy, our Fed-GPL exceeds the method trained from scratch by at least 3.50%, surpassing the full fine-tuning method by 3.01%. Although the naive federated visual prompt learning method Fed-VPL has certain advantages over other traditional methods, it is still constrained by domain shifts posed by multi-center data, which is mitigated via the prompt generator in Fed-GPL. In melanoma classification, we utilize the Area Under the Curve (AUC) to evaluate binary classification. In this task, our method outperforms the competing methods in all domains. Fed-GPL surpasses the best competing method by 17.85%, reaching 83.16%. Notably, the AUC for Fed-ResNet is slightly below 50%, indicating that the model performs no better than random guessing and fails to capture classification-related knowledge in FL. Additionally, Fed-VPL demonstrates superiority over the competing methods with only 38 thousand parameters, showcasing the benefits of fine-tuning in the federated scenario. Figure 3a illustrates the visualization results for the classification tasks. In the convergence curve for the classification task, our method not only outperforms other methods in terms of accuracy but also exhibits advantages in convergence speed. For instance, in melanoma classification, Fed-GPL reaches a high AUC around 15–20 communication rounds and subsequently maintains a stable performance, achieving more than 95% of its final performance observed at 30 rounds. In contrast, the baseline methods, Fed-ResNet and Fed-ViT, converge more slowly and experience greater oscillations during training, requiring substantially more rounds to approach their final performance and exhibiting less stable optimization trajectories.

**Table 1:** Performance evaluation on the classification task

method	Diabetic retinopathy (ACC) *↑*					Melanoma (AUC) *↑*					
	*A*	*E*	*M-1*	*M-2*	*Avg*	*c*	*d*	*g*	*h*	*r*	*Avg*
Fed-ResNet	49.50	71.00	44.50	59.50	56.13	52.44	46.45	49.84	54.90	44.01	49.53
Fed-ViT	54.00	70.50	44.50	59.50	57.13	64.51	70.63	71.63	58.35	49.33	62.89
Fed-ViT-Full	55.00	71.50	45.50	58.50	57.62	73.63	66.41	66.37	60.85	51.66	63.78
Fed-VPL (ours)	53.50	73.50	44.50	59.50	57.75	69.59	64.24	71.18	65.35	56.21	65.31
Fed-GPL (ours)	**58.50**	**74.50**	**48.50**	**61.00**	**60.63**	**82.45**	**85.44**	**83.09**	**85.58**	**79.29**	**83.16**

We used ACC and AUC for multi-class and binary classification tasks. The table presents the performance of the corresponding metrics across different domains, along with the average performance. The best results are highlighted in bold.

We present a quantitative analysis of the segmentation tasks in Table 2. Fed-GPL demonstrates superior performance in both polyp and prostate segmentation tasks, as indicated by the Dice Similarity Coefficient (DSC) and Intersection over Union (IoU) metrics. Regarding 95th percentile Hausdorff Distance (HD95), our method excels in most domains and in terms of average performance, although it is slightly outperformed by some methods in the *C-300*, *ETIS*, *BMC*, and *BID* domains. This can be attributed to the fact that the loss function primarily emphasizes DSC, which does not necessarily ensure optimal HD95 performance. Among the comparison methods, the segmentation performance of the pre-trained SAM model with box prompts is subpar, with the DSC score being 12.57% lower in the polyp task and 5.50% lower in the prostate task compared to the fine-tuning methods. While Fed-U-Net and Fed-DeepLabV3+, both utilizing a ResNet-50 image encoder, show some improvement, all metrics remain inferior to the SAM fine-tuning method due to limitations related to data size and training rounds. In the SAM-based fine-tuning approach, our proposed Fed-VPL performs comparably to Fed-SAM, with the DSC and IoU gap remaining within 1% for most tasks. For HD95, the average value of Fed-SAM is lower than that of Fed-VPL, indicating slightly better segmentation performance. The additional trainable parameters of Fed-SAM (image and prompt encoders) yield limited improvements, and both approaches are still influenced by domain feature shifts. The visualization results of the segmentation task are presented in Fig. 3. In Fig. 3a, although Fed-U-Net and Fed-DeepLabV3+ are fine-tuned from pre-trained models, the initial DSC of both methods remains relatively low due to the addition of extra channels, and both methods converge to a lower value. In contrast, SAM-based fine-tuning methods start at a higher point and converge more quickly. Additionally, Fig. 3b showcases specific examples from polyp and prostate segmentation. The unfine-tuned SAM model produces relatively coarse boundaries when segmenting medical images. While Fed-DeepLabV3+ and Fed-U-Net show improvements in DSC, their segmentation boundaries remain less smooth. Among the SAM-based fine-tuning methods, Fed-SAM and Fed-VPL yield similar results, with Fed-GPL further enhancing segmentation precision.

**Table 2:** Performance evaluation on the segmentation task

method	metric	Polyp						Prostate						
		*C-300*	*C-Cli*	*C-Col*	*ETIS*	*Kvasir*	*Avg*	*RUN*	*BMC*	*HCR*	*UCL*	*BID*	*HK*	*Avg*
Fed-U-Net	DSC *↑*	95.14	90.32	84.19	87.39	85.53	88.52	93.11	90.66	92.03	88.49	91.76	88.36	90.74
	IoU *↑*	91.16	83.38	77.58	79.87	79.87	82.37	87.16	92.97	85.27	79.50	84.87	79.30	84.85
	HD95 *↓*	6.37	18.18	29.94	35.04	37.46	25.39	5.34	6.83	5.11	7.37	5.33	5.36	5.89
Fed-DeepLabV3+	DSC *↑*	83.64	89.38	78.52	74.32	92.98	83.77	89.60	89.86	92.46	88.59	88.67	80.04	88.20
	IoU *↑*	73.50	81.13	65.78	62.64	87.47	74.10	81.23	81.64	86.01	79.64	79.69	67.42	79.27
	HD95 *↓*	67.05	18.47	39.77	264.81	15.72	81.16	6.24	5.88	4.71	8.08	6.04	19.69	8.44
SAM	DSC *↑*	90.09	82.69	81.97	57.34	78.87	78.19	89.32	87.07	85.85	89.11	86.21	85.28	87.14
	IoU *↑*	82.18	71.75	69.70	40.47	65.63	65.95	80.84	77.40	77.13	80.51	76.05	74.61	77.76
	HD95 *↓*	6.23	12.81	16.92	291.40	34.66	72.40	24.81	6.75	6.55	6.47	7.07	5.86	5.92
Fed-SAM	DSC *↑*	94.68	91.40	91.59	92.36	83.80	90.76	94.66	92.17	93.39	91.85	92.48	92.64	92.86
	IoU *↑*	90.10	85.41	84.73	86.11	77.94	84.86	90.04	86.00	87.67	85.38	86.27	86.56	86.99
	HD95 *↓*	5.82	15.80	12.71	25.96	46.68	21.40	4.45	9.28	4.54	4.89	6.14	4.43	5.62
Fed-VPL (ours)	DSC *↑*	94.48	92.19	90.91	92.16	85.05	90.96	94.43	92.02	93.06	92.21	92.10	92.01	92.64
	IoU *↑*	89.70	86.36	83.69	85.91	77.91	84.71	89.60	85.68	87.09	85.89	85.63	85.39	86.55
	HD95 *↓*	6.24	12.64	20.99	30.40	45.71	23.20	4.75	10.79	4.81	4.36	5.96	4.50	5.86
Fed-GPL (ours)	DSC *↑*	**95.24**	**93.99**	**91.98**	**94.61**	**93.46**	**93.86**	**95.41**	**93.71**	**94.37**	**93.51**	**92.96**	**93.88**	**93.97**
	IoU *↑*	*90.94*	*89.01*	*85.39*	*90.05*	*87.91*	*88.66*	*91.32*	*88.63*	*89.37*	*88.02*	*87.23*	*88.57*	*88.86*
	HD95 *↓*	5.95	9.31	12.61	29.06	33.90	18.17	4.19	6.12	4.46	4.21	5.97	3.79	4.79

We evaluated the two segmentation dataset tasks using DSC, IoU, and HD95. The best DSC performance is highlighted in bold, the best IoU performance is in italics, and the best HD95 performance is underlined. Notably, SAM refers to utilizing the box-prompt-based SAM model for prediction, with no parameters involved in training.

For both diabetic retinopathy (Table S7) and melanoma classification (Table S8), the models were evaluated using imbalance-aware metrics. In diabetic retinopathy classification, Fed-ViT-Full showed the lowest performance with a Macro-F1 score of 0.1618, struggling to detect severe (Class 4) and proliferative diabetic retinopathy (Class 5). Fed-VPL performed slightly better with a Macro-F1 of 0.1998, but still faced challenges with the more severe categories. Fed-GPL outperformed the other models, achieving a Macro-F1 score of 0.3317 and demonstrating more balanced performance, particularly for moderate (Class 3) and mild (Class 2) stages. In melanoma classification, Fed-ViT-Full had a low Macro-F1 score of 0.4519, with an F1 score of 0.0190 for melanoma detection (Class 2). Fed-VPL improved with a Macro-F1 of 0.5562, but Fed-GPL excelled with a Macro-F1 score of 0.7314 and an F1 score of 0.5638 for melanoma detection, significantly outperforming the other models. These results were further corroborated by confusion matrices and ROC/PR curves (Fig. S2). Fed-GPL consistently showed superior performance across all metrics, particularly in handling class imbalance, providing better precision-recall trade-offs for challenging minority classes. The PR curve for Fed-GPL achieved an AP of 0.6720, outperforming Fed-ViT-Full (AP of 0.3075) and Fed-VPL (AP of 0.3684), highlighting its advantage in balancing precision and recall. Regarding Fig. 3c, we can observe the attention heatmaps for different models on medical image datasets, generated using Grad-CAM. By comparing the original images, ground truth, and attention heatmaps generated by various models (ViT, Fed-ViT-Full, Fed-VPL, Fed-GPL), we can clearly see how Fed-GPL focuses on more relevant regions of the image. The heatmaps highlight the regions the model focuses on, with Fed-GPL showing more stable and focused localization of key regions, especially when identifying smaller areas like melanoma. In conclusion, Fed-GPL emerges as the best-performing model for both diabetic retinopathy and melanoma classification, particularly in tasks with class imbalance, providing better minority class recognition and overall performance.

### Ablation study on prompt

**Fig. 4:**
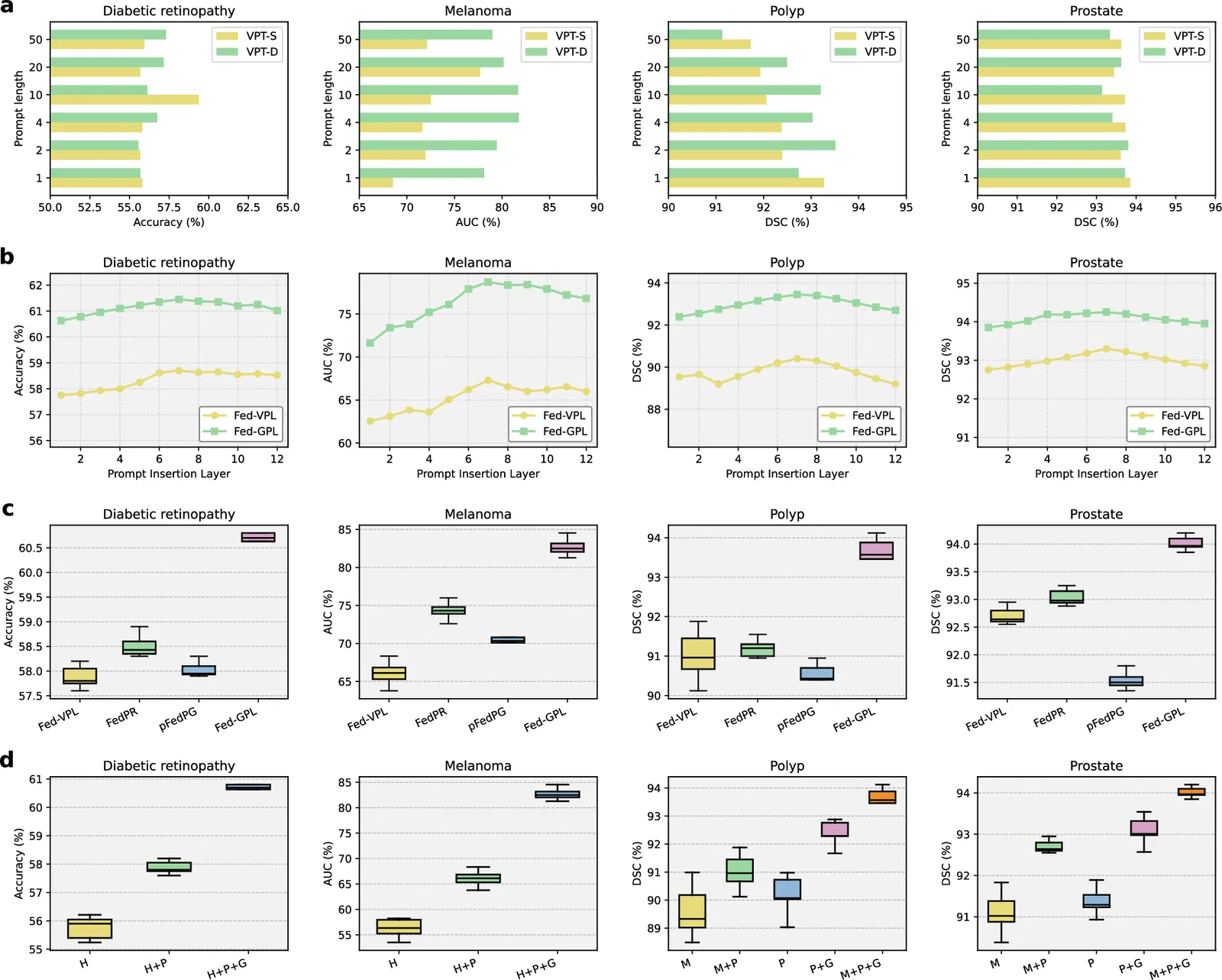
Comprehensive Analysis of Fed-GPL and Baseline Performance. **a** Sensitivity to Prompt Length: Performance of VPT-S and VPT-D across different prompt lengths on four medical imaging tasks. **b** Impact of Prompt Insertion Layer: Comparison of Fed-VPL and Fed-GPL with prompts inserted at different ViT layers. **c** Comparison with State-of-the-Art Methods: Box plots comparing Fed-VPL, FedPR, pFedPG, and Fed-GPL over multiple runs. **d** Component-level Ablation Analysis: Component-wise ablation results for classification and segmentation, showing the contribution of each module.

To comprehensively evaluate the Fed-GPL framework, we conduct a series of analyses on prompt configurations, architectural influence, and component efficacy, as illustrated in Fig. 4. First, we investigate the sensitivity of performance to prompt length and type (VPT-Shallow vs. VPT-Deep) across all tasks in Fig. 4a. For classification tasks, performance typically peaks at intermediate prompt lengths (e.g., length 10 for DR and length 4 for Melanoma), as excessively long prompts may introduce redundant information. In contrast, for segmentation tasks like Prostate, shorter prompts (length 1) prove to be more parameter-efficient while maintaining high DSC scores.

The impact of the prompt insertion layer is further explored in Fig. 4b. By comparing Fed-VPL and Fed-GPL across the 12 blocks of the ViT backbone, we observe that Fed-GPL consistently maintains superior performance across all depths. Notably, the performance gap between Fed-GPL and the baseline widens as prompts are inserted into deeper layers, suggesting that our proposed generator effectively captures high-level semantic features required for complex medical imaging tasks.

Figure 4c provides a quantitative comparison between Fed-GPL and state-of-the-art methods including Fed-VPL, FedPR^54^, and pFedPG^55^. The distribution of results over multiple independent runs indicates that Fed-GPL not only achieves higher mean accuracy and DSC across all datasets but also exhibits tighter variance, demonstrating its robustness against heterogeneous data distributions.

Finally, we conduct component-level ablations to isolate the effects of the classification head/mask decoder (H/M), global prompts (P), and our proposed prompt generator (G), with results summarized in Fig. 4d. For classification, the full configuration (H+P+G) significantly outperforms training the head alone or with fixed prompts. In segmentation tasks, we consider five configurations: (i) mask decoder only (M), (ii) decoder with global prompt (M+P), (iii) global prompt only (P), (iv) prompt generator only (G), and (v) the complete Fed-GPL (M+P+G). These results underscore that the synergy between the task-specific decoder and our dynamic prompt generator is essential for achieving optimal segmentation masks, validating the efficacy of each designed component.

### Privacy leakage analysis

**Fig. 5:**
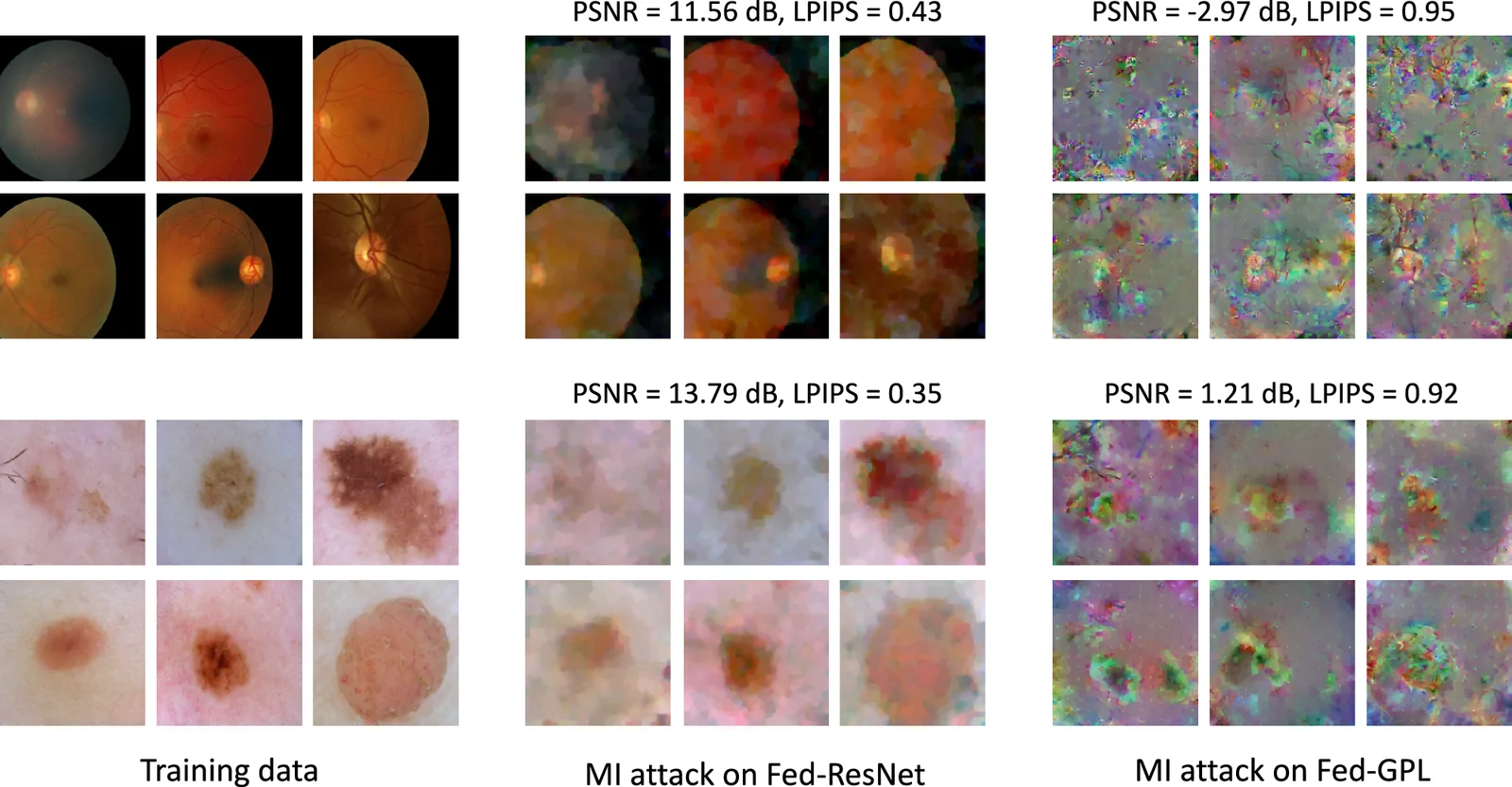
Visualization results of model inversion attacks on the classification task. The first column shows training samples, followed by reconstructed images obtained from model inversion attacks against Fed-ResNet and Fed-GPL, respectively. Reconstruction quality is evaluated by Peak Signal-to-Noise Ratio (PSNR) and Learned Perceptual Image Patch Similarity (LPIPS). Higher PSNR and lower LPIPS indicate stronger privacy leakage.

Traditional federated learning has been proven vulnerable to model inversion (MI) attacks^56,57^, where attackers attempt to recover original training data by exploiting gradients or shared model parameters uploaded by local clients. In our study, we apply a representative MI attack^57^ to both traditional federated learning and the proposed Fed-GPL on the classification task, as illustrated in Fig. 5. From a visual inspection perspective, the MI attack on Fed-ResNet is able to recover coarse structures and partial semantic features that resemble the original training images, whereas the reconstructed results obtained from Fed-GPL exhibit highly degraded and noisy patterns, with no discernible semantic content. Quantitatively, we evaluate reconstruction quality using both Peak Signal-to-Noise Ratio (PSNR) and Learned Perceptual Image Patch Similarity (LPIPS). As shown in Fig. 5, MI attacks on Fed-ResNet yield consistently higher PSNR and lower LPIPS values, indicating stronger pixel-level and perceptual similarity to the original images. In contrast, Fed-GPL results in substantially lower PSNR and significantly higher LPIPS, reflecting poor reconstruction fidelity and weak perceptual alignment. These results reveal that Fed-GPL effectively hinders attackers from recovering meaningful visual information about the original training data from the shared model updates. Overall, by limiting the exposure of sensitive model parameters and decoupling instance-level information through generative prompt learning, Fed-GPL substantially mitigates privacy leakage under MI attacks, demonstrating improved resistance to reconstruction-based privacy threats in federated learning.

### Low-resource performance

We also evaluate the performance of Fed-GPL and the comparison methods in low-resource scenarios. For the classification task in Table 3, the amount of local data on each client $| {\mathcal{D}}|$ is set to 10, 25, 50, 100, 200, 400, and 800, with ACC and AUC used as evaluation metrics across two datasets, respectively. For the segmentation task in Table 4, the data size $| {\mathcal{D}}|$ is set to 2, 5, 10, 15, 20, 30, and 40, using DSC and HD95 as evaluation metrics (as IoU and DSC are numerically correlated, IoU is no longer used for comparison). As expected, performance improves across all methods with increasing local training data. In the classification task, our method performs slightly worse than Fed-ViT-Full on the DR dataset with 800 training samples, but leads other methods in all other settings. Moreover, Fed-GPL shows lower sensitivity to data volume compared to others, making it particularly advantageous in low-resource scenarios. Notably, even with very limited training data ($| {\mathcal{D}}|$ = 10 or 25), our method maintains great classification performance, highlighting the necessity of integrating fine-tuning paradigms into federated learning. Fed-GPL consistently surpasses Fed-VPL in various settings, demonstrating the effectiveness of prompt generation for mitigating domain shifts. In the segmentation task, our method also demonstrates advantages. With only 2 training samples per client, Fed-GPL outperforms Fed-DeepLabV3+ by 45.26% and 39.85% in DSC on the two datasets, respectively. SAM-based fine-tuning methods achieve relatively high DSC and HD95 in low-resource scenarios, yet Fed-GPL consistently maintains a 2% and 1% lead across different settings. Notably, in our comparative experiments, we have mentioned that Fed-VPL slightly underperforms compared to Fed-SAM. However, in low-resource scenarios ($| {\mathcal{D}}|$ = 2 or 5), Fed-VPL surpasses Fed-SAM across all metrics, highlighting the data efficiency of prompt learning. Moreover, the HD95 of Fed-GPL fails to reach optimal performance in some settings ($| {\mathcal{D}}| =2,15$ in polyp segmentation and $| {\mathcal{D}}| =2$ in prostate segmentation), which can also be attributed to the design of the objective function. Nonetheless, our method still achieves promising results in low-resource settings, addressing data scarcity in FL.

**Table 3:** Performance comparison for classification task in the low-resource scenario

DR	metric	local training data size						
		*10*	*25*	*50*	*100*	*200*	*400*	*800*
Fed-ResNet	ACC *↑*	52.19 ± 0.06	53.01 ± 0.12	53.56 ± 0.06	54.94 ± 0.19	55.12 ± 0.13	56.13 ± 0.06	56.50 ± 0.00
Fed-ViT	ACC *↑*	49.13 ± 1.13	51.63 ± 1.13	55.62 ± 1.01	56.87 ± 0.00	56.93 ± 0.06	57.06 ± 0.06	57.06 ± 0.06
Fed-ViT-Full	ACC *↑*	49.12 ± 0.01	52.44 ± 0.19	53.69 ± 0.06	55.50 ± 0.32	57.62 ± 0.12	57.62 ± 0.12	58.37 ± 0.12
Fed-VPL (ours)	ACC *↑*	52.00 ± 0.38	53.42 ± 0.28	56.81 ± 0.06	56.92 ± 0.12	57.06 ± 0.06	57.75 ± 0.06	58.25 ± 0.25
Fed-GPL (ours)	ACC *↑*	**53.88** ± **0.50**	**56.12** ± **0.24**	**57.81** ± **0.31**	**58.31** ± **0.06**	**59.21** ± **0.17**	**60.63** ± **0.06**	**61.12** ± **0.06**
**Melanoma**	metric	local training data size						
		*10*	*25*	*50*	*100*	*200*	*400*	*800*
Fed-ResNet	AUC *↑*	51.92 ± 0.27	51.69 ± 0.64	50.74 ± 1.30	52.18 ± 0.42	51.70 ± 0.11	49.35 ± 0.18	51.52 ± 1.54
Fed-ViT	AUC *↑*	53.52 ± 0.02	51.29 ± 0.00	56.89 ± 0.05	59.57 ± 0.10	61.54 ± 0.09	62.76 ± 0.13	66.14 ± 0.13
Fed-ViT-Full	AUC *↑*	42.03 ± 0.11	39.82 ± 0.05	42.54 ± 0.08	50.57 ± 0.21	57.50 ± 0.16	63.60 ± 0.18	69.85 ± 0.12
Fed-VPL (ours)	AUC *↑*	45.10 ± 0.08	42.28 ± 0.02	46.40 ± 0.05	55.66 ± 0.15	53.59 ± 0.00	65.12 ± 0.16	71.50 ± 0.11
Fed-GPL (ours)	AUC *↑*	**71.87** ± **0.14**	**70.43** ± **0.18**	**72.81** ± **0.00**	**74.87** ± **0.07**	**78.92** ± **0.37**	**82.79** ± **0.37**	**85.25** ± **0.00**

Each experiment is repeated five times. We set the local training data size as 10, 25, 50, 100, 200, 400, and 800. The best results are highlighted in bold, our method outperforms other comparison methods in most settings.

**Table 4:** Performance comparison for segmentation task in low-resource scenario

Polyp	metric	local training data size						
		*2*	*5*	*10*	*15*	*20*	*30*	*40*
Fed-U-Net	DSC *↑*	84.42 ± 0.18	86.77 ± 0.15	87.13 ± 0.57	87.43 ± 0.03	88.07 ± 0.33	88.52 ± 0.15	90.24 ± 0.12
	HD95 *↓*	35.89 ± 0.63	32.49 ± 0.19	28.06 ± 0.42	26.92 ± 0.10	25.32 ± 0.52	25.39 ± 0.17	24.20 ± 0.24
Fed-DeepLabV3+	DSC *↑*	45.62 ± 0.18	69.25 ± 0.30	72.78 ± 0.12	73.01 ± 0.43	78.91 ± 0.80	83.49 ± 0.28	83.07 ± 1.16
	HD95 *↓*	311.97 ± 10.03	214.87 ± 3.92	108.00 ± 6.34	94.53 ± 1.27	79.28 ± 2.74	81.16 ± 3.78	77.12 ± 2.35
Fed-SAM	DSC *↑*	88.51 ± 0.01	88.71 ± 0.59	89.84 ± 0.22	90.74 ± 0.06	90.45 ± 0.17	90.76 ± 0.02	90.56 ± 0.12
	HD95 *↓*	27.21 ± 0.08	24.00 ± 0.44	23.21 ± 0.10	20.71 ± 0.06	23.70 ± 0.36	21.40 ± 0.03	22.74 ± 0.24
Fed-VPL (ours)	DSC *↑*	89.51 ± 0.07	89.11 ± 0.12	88.97 ± 0.40	90.44 ± 0.28	90.90 ± 0.31	90.98 ± 0.02	91.26 ± 0.18
	HD95 *↓*	24.80 ± 0.02	30.99 ± 0.23	27.07 ± 0.41	22.19 ± 0.32	21.34 ± 0.29	23.23 ± 0.04	23.35 ± 0.20
Fed-GPL (ours)	DSC *↑*	**90.78** ± **0.36**	**90.92** ± **0.64**	**92.00** ± **0.52**	**91.97** ± **0.27**	**92.64** ± **0.10**	**93.51** ± **0.34**	**94.11** ± **0.26**
	HD95 *↓*	26.39 ± 0.67	23.01 ± 0.26	21.99 ± 0.20	23.54 ± 0.82	21.31 ± 0.92	21.11 ± 1.06	18.28 ± 0.34
**Prostate**	metric	local training data size						
		*2*	*5*	*10*	*15*	*20*	*30*	*40*
Fed-U-Net	DSC *↑*	70.46 ± 0.56	75.85 ± 0.15	81.54 ± 0.16	84.28 ± 0.07	87.36 ± 0.12	90.60 ± 0.14	89.60 ± 1.03
	HD95 *↓*	86.59 ± 2.35	46.81 ± 0.21	28.07 ± 0.22	15.97 ± 0.15	8.99 ± 0.05	5.89 ± 0.09	6.13 ± 0.52
Fed-DeepLabV3+	DSC *↑*	51.89 ± 0.14	65.37 ± 0.33	67.10 ± 0.06	83.66 ± 0.18	86.03 ± 0.05	88.17 ± 0.03	89.34 ± 0.35
	HD95 *↓*	152.20 ± 2.30	130.97 ± 1.59	127.73 ± 0.57	28.62 ± 0.15	15.83 ± 0.07	8.40 ± 0.04	6.25 ± 0.28
Fed-SAM	DSC *↑*	91.56 ± 0.02	91.88 ± 0.42	92.51 ± 0.05	92.73 ± 0.03	92.85 ± 0.59	93.09 ± 0.23	92.97 ± 0.23
	HD95 *↓*	6.32 ± 0.11	6.55 ± 0.29	5.93 ± 0.07	5.50 ± 0.11	5.55 ± 0.17	5.58 ± 0.04	5.50 ± 0.14
Fed-VPL (ours)	DSC *↑*	**91.94** ± **0.04**	92.27 ± 0.04	92.01 ± 0.05	92.64 ± 0.13	92.77 ± 0.03	92.75 ± 0.11	92.98 ± 0.10
	HD95 *↓*	6.16 ± 0.03	5.94 ± 0.03	5.68 ± 0.02	5.80 ± 0.10	5.66 ± 0.11	5.76 ± 0.10	5.46 ± 0.09
Fed-GPL (ours)	DSC *↑*	91.74 ± 0.23	**93.18** ± **0.07**	**93.38** ± **0.11**	**93.53** ± **0.01**	**93.83** ± **0.17**	**93.85** ± **0.12**	**93.84** ± **0.03**
	HD95 *↓*	6.32 ± 0.19	5.42 ± 0.05	5.30 ± 0.38	5.22 ± 0.02	5.10 ± 0.15	4.79 ± 0.07	4.88 ± 0.02

Each experiment is repeated five times. We set the local training data size as 2, 5, 10, 15, 20, 30, and 40. We evaluate different methods using DSC and HD95, with the best DSC results highlighted in bold and the best HD95 results underlined. Our method outperforms the others under most settings.

### Effect of prompt generator

The prompt generator is the most critical component of Fed-GPL, mitigating the problem of domain feature shift in cross-hospital scenarios. We allow each client to train the visual prompts on their local datasets and test the personalized prompts across multiple domains. The results are shown in Fig. S1. In the four heatmaps, we observe that the diagonal blocks are often the darkest in each column (excluding Fed-GPL and Fed-VPL). This indicates that the prompts fine-tuned on a specific domain perform well in the corresponding domain but fail to adapt to the styles of other domains. At the same time, the average performance of Fed-VPL is relatively low, even falling behind some personalized prompts in the classification tasks. This further reflects the feature shift phenomenon in cross-domain prompt tuning, which limits the generalization capability of federated prompt tuning. The introduction of the prompt generator effectively alleviates this phenomenon. Fed-GPL outperforms the optimal personalized prompt and Fed-VPL in most domains, as well as in average performance. Compared with Fed-VPL, Fed-GPL surpasses it by 2.13%, 17.85%, 2.90%, and 1.33% on four datasets, respectively. Fed-GPL shows great generalization ability, which is achieved by the instance-level prompt generation. Fed provides an appropriate prompt for each sample (patient), enhancing the performance of the single sample in downstream tasks. This, in turn, contributes to improving the overall performance of the global model and guarantees the generalization ability.

To further evaluate cross-center generalization capability of prompt generator, we conduct Leave-One-Hospital-Out (LOHO) experiments, where data from one hospital are held out as an unseen test domain, and the remaining hospitals collaboratively train the model in a federated manner. This setting simulates real-world scenarios where models are deployed to previously unseen medical centers. We perform LOHO evaluation on melanoma classification and polyp segmentation as representative tasks, and the corresponding results are reported in Table S1 and Table S2 in the Supplementary Material. The results indicate that Fed-GPL consistently outperforms baseline federated and prompt-based methods across held-out hospitals, achieving the highest AUC for melanoma classification (76.44%) and the best DSC for polyp segmentation (92.05%), as shown in Tables S1 and S2, respectively. In contrast, methods based on global prompts or full fine-tuning show more pronounced performance degradation on unseen centers, highlighting their limited generalization ability. These findings further validate that the instance-level prompt generation mechanism in Fed-GPL enhances robustness to cross-center distribution shifts, demonstrating superior adaptability to heterogeneous data distributions in multi-center medical settings.

Besides, we further demonstrate the contribution of the generator component to performance improvement through an ablation study in Fig. 4(d). The results show that adding the generator consistently boosts performance across tasks. For example, in Diabetic Retinopathy, the accuracy improves from 55.24% with just the classification head to 60.63% with the full setup. Similar improvements are observed in Melanoma, Polyp, and Prostate, highlighting the generator’s key role in enhancing model performance and adaptability to diverse data distributions.

### Hyperparameter analysis

Table S10 presents the results of hyperparameter sensitivity and ablation studies across four tasks. For learning rate, the best results are achieved with 1 × 10^−6^, providing the highest performance for DR (60.63%) and Melanoma (83.16%). Higher learning rates, such as 5 × 10^−5^, result in lower performance, particularly for DR, indicating that a smaller learning rate helps avoid overshooting and aids convergence. In terms of local training epochs, 2 epochs yield the best overall performance, with DR, Melanoma, Polyp, and Prostate all showing peak metrics. Training for 3 or more epochs does not significantly improve results and increases computational cost, making 2 epochs the optimal choice. For prompt initialization, both zero-start and random initialization strategies produce similar outcomes, but zero-start initialization slightly outperforms random initialization, especially for DR and Melanoma, leading to the best results. Finally, for number of generator heads, 12 heads give the best performance for DR, Melanoma, and Polyp. Although using 18 heads improves the Dice score for Polyp and Prostate, the computational cost increases, so 12 heads provide a better trade-off between performance and efficiency.

### System cost analysis

This section compares the system and communication costs of different federated learning (FL) frameworks, focusing on classification and segmentation tasks across various datasets. Table 5 summarizes key metrics such as trainable parameters, communication costs, training time, and rounds to convergence. Fed-VPL, our approach, significantly reduces communication and training costs by minimizing the number of trainable parameters. For instance, in the *diabetic retinopathy* task, Fed-VPL requires only 4.60 × 10^4^ bytes per round, compared to the 3.43 × 10^8^ bytes of Fed-ViT-Full. Its training time per round is also faster, typically under 200 seconds. Fed-GPL, while having slightly higher communication and computation costs, offers improved generalization, with similar training times to Fed-VPL. For example, in the *diabetic retinopathy* task, Fed-GPL’s total training time over 30 rounds (6097.65 seconds) is comparable to Fed-VPL (5612.62 seconds), but it provides better generalization.

**Table 5:** System and communication cost comparison across datasets (single-machine FL simulation on NVIDIA RTX A40; communication bytes assume fp32, i.e., 4 bytes/parameter)

Classification task						
Metric	*diabetic retinopathy*			*melanoma*		
	Fed-ViT-Full	Fed-VPL	Fed-GPL	Fed-ViT-Full	Fed-VPL	Fed-GPL
Backbone params	8.58 × 10^7^	8.58 × 10^7^	8.58 × 10^7^	8.58 × 10^7^	8.58 × 10^7^	8.58 × 10^7^
Trainable params	8.58 × 10^7^	1.15 × 10^4^	7.09 × 10^6^	8.58 × 10^7^	3.84 × 10^4^	2.95 × 10^7^
Percentage	100%	0.013%	8.26%	100%	0.045%	34.38%
Uplink bytes/round	3.43 × 10^8^	4.60 × 10^4^	2.84 × 10^7^	3.43 × 10^8^	1.54 × 10^5^	1.18 × 10^8^
Downlink bytes/round	3.43 × 10^8^	4.60 × 10^4^	2.84 × 10^7^	3.43 × 10^8^	1.54 × 10^5^	1.18 × 10^8^
Total bytes (30 rounds)	2.06 × 10^10^	2.76 × 10^6^	1.70 × 10^9^	2.06 × 10^10^	9.22 × 10^6^	7.08 × 10^9^
Time/round (s)	194.35	187.09	203.26	204.89	197.97	218.80
Total time (15 rounds)	2883.06	2792.58	3057.09	3085.92	2923.95	3261.46
Total time (30 rounds)	5830.38	5612.62	6097.65	6130.45	5926.10	6563.98
Rounds to 95% final	20	13	14	23	12	15
**Segmentation task**						
Metric	*polyp*			*prostate*		
	Fed-SAM	Fed-VPL	Fed-GPL	Fed-SAM	Fed-VPL	Fed-GPL
Backbone params	9.37 × 10^7^	9.37 × 10^7^	9.37 × 10^7^	9.37 × 10^7^	9.37 × 10^7^	9.37 × 10^7^
Trainable params	9.37 × 10^7^	5.24 × 10^6^	2.08 × 10^7^	9.37 × 10^7^	4.11 × 10^6^	6.14 × 10^6^
Percentage	100%	5.59%	22.21%	100%	4.39%	6.55%
Uplink bytes/round	3.75 × 10^8^	2.10 × 10^7^	8.32 × 10^7^	3.75 × 10^8^	1.64 × 10^7^	2.46 × 10^7^
Downlink bytes/round	3.75 × 10^8^	2.10 × 10^7^	8.32 × 10^7^	3.75 × 10^8^	1.64 × 10^7^	2.46 × 10^7^
Total bytes (30 rounds)	2.25 × 10^10^	1.26 × 10^9^	4.99 × 10^9^	2.25 × 10^10^	9.84 × 10^8^	1.48 × 10^9^
Time/round (s)	81.80	80.71	85.86	79.61	77.55	83.08
Total time (15 rounds)	1220.83	1213.21	1266.63	1224.29	1171.54	1251.34
Total time (30 rounds)	2454.04	2421.15	2575.83	2388.15	2326.54	2492.41
Rounds to 99% final	17	7	13	16	9	11

Table S9 in supplementary materials further illustrates the per-client distribution of trainable parameters, reinforcing the efficiency of Fed-VPL in reducing the computational load while maintaining competitive performance. Overall, Fed-VPL is more cost-efficient, while Fed-GPL offers enhanced generalization without a significant increase in training time. Both methods outperform Fed-ViT-Full in terms of efficiency.

## Discussion

Federated learning in multi-institutional medical image analysis is constrained by communication overhead, data scarcity, and heterogeneity. To address these challenges, we introduce Fed-GPL, a universal and efficient federated prompt learning framework that improves generalization performance in multi-center scenarios.

Our approach incorporates foundation models with parameter-efficient prompt learning into federated learning, which reduces the number of trainable parameters and communication overhead during collaborative training, while leveraging pre-trained representations to alleviate data scarcity. In addition, we designed a cross-attention-based prompt generator to mitigate feature shifts in multi-center medical data. It utilizes global prompts to encapsulate task-specific knowledge, which is then queried using image embeddings to produce tailored prompts for each input sample. By generating personalized prompts, Fed-GPL optimizes FM performance on individual instances, further boosting its adaptation capability. By explicitly coupling global medical priors encoded in foundation models with patient-specific visual features, the prompt generator enables instance-level adaptation and helps detect subtle pathological patterns that may be overlooked in purely global or purely local optimization settings. In doing so, Fed-GPL effectively balances cross-domain generalization and instance-level personalization, thereby enhancing robustness, interpretability, and clinical applicability in real-world federated medical scenarios. Comprehensive experiments were conducted on medical classification and segmentation tasks using four datasets to investigate the universal performance of Fed-GPL in multi-center scenarios. The experimental results demonstrate that our method consistently outperforms baseline methods and other fine-tuning approaches across the majority of experimental settings and datasets with less than 5% of the parameters.

In Fed-GPL, the integration of large-scale vision foundation models provides a principled foundation for addressing key federated learning challenges, beyond merely improving standalone diagnostic performance. Vision foundation models like ViT and SAM, pre-trained on extensive datasets such as ImageNet, possess robust feature extraction capabilities, allowing them to effectively capture meaningful features from diverse medical image types, including fundus photography, dermoscopic images, endoscopic images, and MRI scans. These models excel at identifying complex image features and structural information, providing superior initial conditions for downstream tasks such as lesion detection, organ segmentation, and image classification. Moreover, the strong transferability of foundation models facilitates rapid adaptation to downstream tasks through prompt learning with minimal labeled data, directly mitigating data scarcity in federated medical settings. Additionally, prompt learning requires only a small number of trainable parameters, which reduces communication costs in collaborative training processes and enhances overall training efficiency. This parameter-efficient design is particularly suitable for federated learning, where communication efficiency is a critical constraint. Finally, the strong cross-task adaptability of these foundation models makes them highly effective tools for addressing complex image challenges in medical AI. Building upon these strengths, Fed-GPL incorporates a novel cross-attention-based prompt generator to fully exploit the capabilities of vision foundation models in the federated medical setting. Rather than relying solely on static global prompts, the prompt generator dynamically fuses global task knowledge with patient-specific visual features extracted from individual patch embeddings, producing instance-adaptive prompts that guide the foundation models to attend to clinically relevant regions and features for each input case. This design explicitly targets the heterogeneity challenge in federated learning by enabling fine-grained instance-level adaptation without requiring heterogeneous client models. By tailoring prompts to the unique characteristics of each medical image, this design effectively mitigates feature shifts caused by variations in imaging devices, acquisition protocols, and patient demographics across participating hospitals, while preserving the advantages of global knowledge sharing. We anticipate our study to 1) enable a communication-efficient and generalized multi-center federated learning framework in data-scarce scenarios, and 2) provide insights for foundational model-based personalized healthcare from the perspective of customized prompt generation.

Although Fed-GPL demonstrates strong generalization performance in low-resource multi-institutional medical image analysis, it also has certain limitations. Firstly, the complexity of the prompt generator in our method increases with the length of the prompts. For example, in the case of the prompt generator in SAM, when using the VPT-Deep mechanism and a prompt length of 10, the prompt generator’s parameter reaches 78 M, comparable to the parameter size of full fine-tuning SAM. Therefore, we must balance communication overhead and model performance when selecting prompt configurations. Secondly, while Fed-GPL mitigates the feature shift problem in multi-center scenarios by introducing the prompt generator, it overlooks the class imbalance between hospitals. This poses a challenge for maintaining the performance consistency of prompt generators during collaboration among hospitals with imbalanced categories. Lastly, Fed-GPL still faces potential privacy leakage risks. The transmission of prompts or the prompt generator between hospitals and the central server has been proven vulnerable to property inference and membership inference attacks^58^. Additionally, the transmitted prompt-related parameters could be exposed to model theft and misuse. Hence, it is necessary to explore privacy-preserving strategies, such as differential privacy^59,60^ and secure multi-party computation^61,62^.

In the future, the proposed Fed-GPL framework can be further extended to support clinical integration and translational research. One promising direction is multi-modal integration, where complementary clinical information such as textual reports, laboratory indicators, or longitudinal patient records can be incorporated to enhance personalized and robust decision-making. The parameter-efficient design with frozen foundation model backbones also facilitates real-time deployment in clinical environments with limited computational resources and supports scalable collaboration across hospitals with heterogeneous hardware infrastructures. Moreover, the instance-level prompt generation mechanism improves robustness to device and acquisition heterogeneity, which commonly arises from variations in imaging equipment, protocols, and patient populations across medical centers. From a regulatory and ethical perspective, the federated learning paradigm aligns with data protection requirements by keeping raw patient data local, while future work should incorporate additional privacy-preserving and fairness-aware mechanisms to mitigate prompt-related inference risks. Finally, external, multicenter, and prospective clinical validation under real-world workflows will be essential to assess generalization, interpretability, and clinical utility, thereby supporting reliable deployment and accelerating clinical translation.

It’s worth noting that the proposed Fed-GPL framework, like other artificial intelligence algorithms, is a double-edged sword. On the one hand, it enhances the foundation model’s performance on multi-center medical datasets through collaborative prompt generation. On the other hand, this technology can be misused. First, in a distributed environment, we cannot ensure the honesty of all participating hospitals. Some participants might upload malicious model parameters or gradients, severely compromising model performance and convergence efficiency during collaborative training. Therefore, it is critical to introduce effective detection mechanisms to filter out dishonest or harmful contributions. Second, while Fed-GPL demonstrates resilience against model inversion attacks, it remains vulnerable to membership inference and attribute inference attacks on the generator parameters, raising ethical concerns about the privacy and ownership of medical data. To mitigate these privacy risks, we can leverage privacy-preserving techniques, such as differential privacy and secure multi-party computation. Third, Fed-GPL’s reliance on local hospital datasets introduces biases and limits representativeness. This leads to inconsistent model performance across different groups, which can result in unfair decisions for minority populations, exacerbating health disparities. Additionally, the interpretability of prompt learning remains unclear, and no research has yet clarified the mechanisms through which prompt learning enhances foundation model performance, potentially leading to a lack of trust from patients and healthcare providers in Fed-GPL-based decisions. In conclusion, while the proposed Fed-GPL framework holds significant potential for collaborative fine-tuning across hospitals, it is essential to acknowledge the accompanying risks and implement safeguards to ensure ethical and responsible application.

## Methods

### Ethics statement

All experiments in this study were conducted in accordance with the Declaration of Helsinki. No prospective recruitment of human participants or new clinical data collection was performed by the authors. Instead, all analyses were based exclusively on previously released public medical imaging datasets that had been de-identified or anonymized prior to public dissemination, and our work only involved secondary analysis of such data. These datasets have been widely used in prior peer-reviewed studies, and their original acquisition, release, and governance were handled by the corresponding dataset providers or source publications. Where publicly available, the reported ethics approvals included, for example, Medical University of Vienna (1804/2017), University of Queensland (2017001223 and 2018000074), Memorial Sloan Kettering Cancer Center (16-974), Hospital Clínic de Barcelona (HCP/2019/0413), and Metro South Health (HREC/17/QPAH/816) for components of the ISIC-related melanoma resources. For the polyp segmentation task, the datasets used in this study were obtained from previously released public benchmark resources. For polyp-related resources associated with Hospital Clínic de Barcelona and the CVC benchmark line, publicly available literature reports that the relevant studies involving human data were approved by the Clinical Research Ethics Committee (CREC) of the Hospital Clínic de Barcelona (HCB), with reference number HCB/2014/1148. For Kvasir-SEG, the source publication states that the study was approved by the Norwegian Privacy Data Protection Authority and that, because the data were fully anonymized, both patient consent and approval from the Regional Committee for Medical and Health Research Ethics were waived. For the remaining public benchmark datasets used in this work, including ophthalmic, endoscopic, and prostate MRI datasets, we relied on the original public releases and corresponding source papers, and restricted our study to secondary analysis of de-identified data only.

### Promptable foundation models

Foundation models are large-scale pre-trained models with millions of parameters, which learn extensive knowledge from large-scale datasets. The primary feature of foundation models lies in their promptability, which means that they can complete various tasks with simple prompts and instructions. This significantly enhances the adaptability of foundation models on numerous downstream tasks across various datasets. In this paper, we primarily adopt two representative large-scale foundation models, Vision Transformer (ViT) and Segment Anything (SAM), for medical image analysis.

#### Promptable Vision Transformer

A classic ViT prompt learning method is Visual Prompt Tuning (VPT)^39^. The core idea of VPT is to add learnable prompts to the input space of the image encoder. In VPT, the backbone of ViT is frozen and only the prompt and the classification head ${\mathcal{H}}$ are trainable. For a classic ViT, the input image is preprocessed and divided into *P* patches, which pass the embedding layer and are added with the position encoder. The given patch embedding ${E}_{0}\in {{\mathbb{R}}}^{P\times d}$ is subsequently concatenated with the classification token *x*_0_ to form the input of the transformer encoder, representing as [*x*_0_, *E*_0_]. In VPT, the *l* continuous *d*-dimension embedding ($P\in {{\mathbb{R}}}^{l\times d}$) is added to the input, resulting in [*x*_0_, *P*, *E*_0_]. The mixed input is fed into *L*-layer encoders and gets the classification token *x*_*L*_, which can be transformed to the final classification result through $y={\mathcal{H}}({x}_{L})$. As the prompt can be added in various layers, VPT is categorized into VPT-Shallow and VPT-Deep. VPT-Shallow only adds prompt *P* to the first layer encoder, and the corresponding output is used as the input of the following layers:

1 $$\begin{array}{l}\begin{array}{l}\left[{x}_{1},{M}_{1},{E}_{1}\right]={I}_{1}(\left[{x}_{0},P,{E}_{0}\right])\\ \left[{x}_{j},{M}_{j},{E}_{j}\right]={I}_{j}(\left[{x}_{j-1},{Q}_{j-1},{E}_{j-1}\right]),j=2,3\ldots L\end{array}\end{array}$$

where ${M}_{j}\in {{\mathbb{R}}}^{l\times d}$ is the feature result in *j*th layer. VPT-Deep adds prompt *P*_*j*_ for each encoder layer *j*, and the overall process can be formulated as follows:

2 $$[{x}_{j},Q,{E}_{j}]={{\mathcal{I}}}_{j}([{x}_{j-1},{P}_{j-1},{E}_{j-1}]),j=1\ldots L$$

where *Q* represents the useless intermediate value. Both methods demonstrate strong performance across most downstream tasks. VPT-Deep, with its relatively larger number of prompt parameters, can achieve better results in certain tasks but at the expense of increased computational cost.

#### Promptable segment anything

SAM consists of image encoder, prompt encoder, and mask decoder. SAM utilizes a pre-trained ViT model as the image encoder to encode high-resolution image inputs. The prompt encoder is a key module that distinguishes SAM from traditional segmentation models. SAM can output different segmentation results for the same input based on varied prompts, rather than providing a fixed segmentation result. Finally, the mask decoder integrates image and prompt embeddings with the output token to produce a mask that aligns with the dimensions of the original image. SAM is a foundation model with learnable prompts. When applied to medical image segmentation, a straightforward approach is to provide SAM with location-aware prompts (e.g., points or boxes) guided by domain-specific knowledge. However, physicians or patients in real-world scenarios often pre-select the target organ for segmentation when using SAM. This type of prompt is treated as known and auxiliary information during the segmentation process.

Consequently, we propose a novel prompt learning framework for SAM with customized and learnable visual prompts in the image encoder. Similar to ViT, the image encoder is primarily composed of 12 stacked transformer blocks. For an input image of size 1024 × 1024, an embedding *E* with dimensions 64 × 64 × *d* is generated after applying patch embedding and position embedding, where *d* is the dimension of patch embedding. Our approach introduces learnable task-specific prompts into the input of the image encoder. Since the patch embedding is three-dimensional, the prompts must align with two of these dimensions to satisfy the requirement of concatenation. Therefore, we define the prompt with dimensions *k* × 64 × *d*, where *k* is the prompt length. After the input passes through the encoder, the prompt-related portion is cropped from the resulting feature map to ensure compatibility with the decoder. During prompt learning, the parameters of both the image encoder and the prompt encoder are frozen. To further enhance segmentation performance on downstream medical tasks, we additionally optimize the mask decoder, which contains only 4 million parameters.

### Federated visual prompt learning

Federated learning achieves collaborative optimization of the global model through local training on clients and model aggregation on the server side. Federated prompt learning alters the type of parameters uploaded by clients, achieving communication efficiency. The objective function of federated prompt learning is given by:

3 $$\mathop{\min }\limits_{{\mathcal{P}}}{\mathcal{L}}({\mathcal{F}}(\cdot ,{\mathcal{P}})):=\frac{1}{N}\mathop{\sum }\limits_{i=1}^{N}{{\mathcal{L}}}_{i}({\mathcal{F}}(\cdot ,{\mathcal{P}}))$$

where ${\mathcal{P}}$ is the trainable and transmitted prompt during the training process. ${\mathcal{L}}$ is the adopted loss function, which differs for different tasks. We first design a naive training strategy to achieve collaborative prompt learning in multi-center scenarios. Fed-VPL integrates prompt learning into federated learning, where only the prompts and the corresponding classification head or mask decoder are trainable, while the backbone of the foundation model remains frozen. Clients locally optimize the prompts and prediction modules and then transmit these parameters to the server. The server collects and aggregates these parameters, distributing the updated prompts and related modules back to the clients for the next round of training. We formulate the local optimization and global aggregation process as follows:

4 $${{\mathcal{P}}}_{i}={{\mathcal{P}}}_{i}-\eta \left(\frac{\partial {{\mathcal{L}}}_{i}}{\partial {{\mathcal{P}}}_{i}}\right),\,{{\mathcal{P}}}^{r+1}=\frac{1}{N}\mathop{\sum }\limits_{i=1}^{N}{{\mathcal{P}}}_{i}^{r}$$

Although Fed-VPL improves the foundation model’s performance on downstream tasks by training only a small portion of parameters, thereby reducing communication costs during federated fine-tuning, its generalization performance under multi-center settings is limited. It fails to meet the prompt requirements of varying data styles under multi-center scenarios.

### Prompt generator

As previously mentioned, both ViT and SAM are promptable vision foundation models. However, manually designed prompts for ViT and SAM are neither practical nor efficient in the medical field. In multi-center scenarios, each institution typically has limited data characterized by distinct stylistic features, making it difficult to produce robust and generalizable prompts through individual training or naive prompt averaging. To address this challenge, we propose a generalized instance-level prompt generation method tailored for multi-institutional collaboration. Specifically, we design a prompt generation mechanism based on cross-attention for each foundation model. This method enhances the global model’s generalization capability by dynamically generating personalized prompts for each input instance. The core idea is to integrate instance-specific information—extracted from the input’s patch embeddings—with general task knowledge encoded in global prompts, thereby producing context-aware and task-relevant prompts that improve performance across diverse hospital datasets.

#### ViT prompt generator

The input of the ViT prompt generator ${\mathcal{G}}$ consists of the global prompt ${{\mathcal{P}}}_{G}\in {{\mathbb{R}}}^{l\times d}$, and the image patch embedding $E\in {{\mathbb{R}}}^{P\times d}$. The output of ${\mathcal{G}}$ is the instance-specific visual prompt ${{\mathcal{P}}}_{s}$ for the following classification tasks. We utilize global prompts to capture task-related, instance-independent knowledge and leverage patch embedding to represent the personalized information unique to each instance. Our core idea is to use *E* as the query and ${{\mathcal{P}}}_{G}$ as the key-value pair, employing cross-attention to capture the relationship between the two elements. We obtain the personalized prompts by calculating the attention scores and performing a weighted summation. Overall, the generation progress can be formulated as follows:

5 $$\begin{array}{l}{\mathcal{Q}}=E{W}^{{\mathcal{Q}}},{\mathcal{K}}={{\mathcal{P}}}_{G}{W}^{{\mathcal{K}}},{\mathcal{V}}={{\mathcal{P}}}_{G}{W}^{{\mathcal{V}}}\\ {{\mathcal{P}}}_{s}={\mathcal{G}}({{\mathcal{P}}}_{G},E)={{\mathcal{P}}}_{G}+softmax(\frac{{\mathcal{Q}}{{\mathcal{K}}}^{T}}{\sqrt{{d}_{k}}}){\mathcal{V}}{W}^{{\mathcal{O}}}\end{array}$$

where *d*_*k*_ is the parameter for scaling, ${W}^{{\mathcal{Q}}},{W}^{{\mathcal{K}}},{W}^{{\mathcal{V}}},{W}^{{\mathcal{O}}}$ are learnable projection matrices in ${\mathcal{G}}$.

#### SAM prompt generator

Similarly, we also design a prompt generation method based on the cross-attention mechanism for SAM. The input of the prompt generator ${\mathcal{G}}$ includes the embedding *E* with the fixed size 64 × 64 × *d* and the global basic prompt ${{\mathcal{P}}}_{G}\in {{\mathbb{R}}}^{k\times 64\times d}$. The cross-attention generator of SAM is the same as Eq. (5). It is worth noting that we have designed a uniform alphabetical representation for the prompt generators of individual ViT and SAM for ease of description, but they still differ somewhat in terms of dimensionality. The inputs of the prompt generator are all inherent embedding results from the inference process, so our method does not introduce any additional computational overhead beyond ${\mathcal{G}}$.

With the prompt generator ${\mathcal{G}}$ and the related parameter *θ*, the prediction process can be formulated as follows:

6 $$\widehat{{\mathcal{Y}}}={\mathcal{F}}({\mathcal{X}},{{\mathcal{G}}}^{R}({{\mathcal{P}}}_{G}^{R},Embed({\mathcal{X}}),\theta ))$$

##### Algorithm 1

Fed-GPL

**Input**: Number of communication rounds *R*, foundation model ${\mathcal{F}}$, prompt generator ${\mathcal{G}}$, global basic prompt ${{\mathcal{P}}}_{G}$, learning rate *η* and local epoch *T*

**Data**: *N* cross-domain datasets ${{\mathcal{D}}}_{1},{{\mathcal{D}}}_{2},\ldots ,{{\mathcal{D}}}_{N}$

**Output**: ${{\mathcal{G}}}^{R}$, ${{\mathcal{P}}}_{G}^{R}$, and ${{\mathcal{H}}}^{R}$ or ${{\mathcal{MD}}}^{R}$

1: **Initialization:** ${{\mathcal{G}}}^{0}$, ${{\mathcal{P}}}_{G}^{0}$, and ${{\mathcal{H}}}^{0}$ or ${{\mathcal{MD}}}^{0}$

2: **Note:** The backbone of foundation model ${\mathcal{F}}$ is frozen throughout training.

3: **for** round *r* = 1, 2, …, *R* **do**

4: # **Clients** (**Local update for** *T* **epochs**)

5: **for** client *i* = 1, 2, …, *N* **do**

6: $$({{\mathcal{X}}}_{i},{{\mathcal{Y}}}_{i})\in {{\mathcal{D}}}_{i}$$

7: Keep foundation model ${\mathcal{F}}$ frozen; only ${\mathcal{G}}$, ${{\mathcal{P}}}_{G}$, and task head are trainable.

8: Embed $${{\mathcal{X}}}_{i}$$ as $$embed({{\mathcal{X}}}_{i})$$;

9: Generate *instance-adaptive prompts*${{\mathcal{P}}}_{s}={\mathcal{G}}({{\mathcal{P}}}_{G},embed({{\mathcal{X}}}_{i}),{\theta }_{i})$;

10: Predict with foundation model ${\widehat{{\mathcal{Y}}}}_{i}={\mathcal{F}}(embed({{\mathcal{X}}}_{i}),{{\mathcal{P}}}_{s})$;

11: Calculate loss ${{\mathcal{L}}}_{i}$ with Eq. (9) or Eq. (10);

12: Update trainable parameters locally: prompt generator ${{\mathcal{G}}}_{i}$, global prompt ${{\mathcal{P}}}_{Gi}$, and classification head ${{\mathcal{H}}}_{i}$ or mask decoder ${{\mathcal{MD}}}_{i}$.

13: $${{\mathcal{P}}}_{Gi}={{\mathcal{P}}}_{Gi}-\eta (\frac{\partial {{\mathcal{L}}}_{i}}{\partial {{\mathcal{P}}}_{Gi}}),\,{\theta }_{i}={\theta }_{i}-\eta (\frac{\partial {{\mathcal{L}}}_{i}}{\partial {\theta }_{i}})$$

14: $${{\mathcal{H}}}_{i}={{\mathcal{H}}}_{i}-\eta (\frac{\partial {{\mathcal{L}}}_{i}}{\partial {{\mathcal{H}}}_{i}})$$ or $${{\mathcal{MD}}}_{i}={{\mathcal{MD}}}_{i}-\eta (\frac{\partial {{\mathcal{L}}}_{i}}{\partial {{\mathcal{MD}}}_{i}})$$

15: **end for**

16: # **Server (Global aggregation once per round)**

17: Receive updated parameters from all *N* clients after *T* local epochs;

18: Update ${{\mathcal{G}}}^{r}$, ${{\mathcal{P}}}_{G}^{r}$, and ${{\mathcal{H}}}^{r}$ or ${{\mathcal{MD}}}^{r}$ with Eq. (8);

19: **Note:** Only global parameters are aggregated; instance-specific prompts are not stored or shared.

20: **end for**

21: **Inference:**

22: Generate personalized prompts on-the-fly using ${{\mathcal{G}}}^{R}$;

23: $$\widehat{{\mathcal{Y}}}={\mathcal{F}}({\mathcal{X}},{{\mathcal{G}}}^{R}({{\mathcal{P}}}_{G}^{R},Embed({\mathcal{X}}),\theta ))$$

### Federated generative prompt learning

To address the generalization issue in federated prompt learning, we propose Fed-GPL, which introduces an instance-level prompt generator to replace the global prompts in Fed-VPL. During the local training process, clients first embed ${\mathcal{X}}$ to obtain the image patch embedding $Embed({\mathcal{X}})$, which is further fed into the prompt generator to generate instance-level prompts ${{\mathcal{P}}}_{s}$. The prompts are concatenated with the corresponding $Embed({\mathcal{X}})$ and fed into the following network of the foundation models. The adjusted local optimization objective function is shown as follows:

7 $$\mathop{\min }\limits_{{{\mathcal{P}}}_{G},\theta }{{\mathbb{E}}}_{({\mathcal{X}},{\mathcal{Y}})\in {{\mathcal{D}}}_{i}}{{\mathcal{L}}}_{i}({\mathcal{F}}({\mathcal{X}},{\mathcal{G}}({{\mathcal{P}}}_{G},Embed({\mathcal{X}}),\theta )),{\mathcal{Y}})$$

Similarly, the server is primarily responsible for parameter aggregation and broadcasting. We formulate the overall optimization and aggregation steps as follows:

8 $$\begin{array}{l}{{\mathcal{P}}}_{Gi}={{\mathcal{P}}}_{Gi}-\eta \left(\frac{\partial {{\mathcal{L}}}_{i}}{\partial {{\mathcal{P}}}_{Gi}}\right),\,{\theta }_{i}={\theta }_{i}-\eta \left(\frac{\partial {{\mathcal{L}}}_{i}}{\partial {\theta }_{i}}\right)\\ {{\mathcal{P}}}_{G}^{r+1}=\frac{1}{N}\mathop{\sum }\limits_{i=1}^{N}{{\mathcal{P}}}_{Gi}^{r},\,{\theta }^{r+1}=\frac{1}{N}\mathop{\sum }\limits_{i=1}^{N}{\theta }_{i}^{r}\end{array}$$

We present the whole process of Fed-GPL in Algorithm 1. It is worth noting that both methods require training and aggregating the classification head ${\mathcal{H}}$ and mask decoder ${\mathcal{MD}}$. However, due to the excessive number of symbols, we did not explicitly display them in the formulas and algorithms.

### Loss function

Specifically, for the classification task, we apply the cross-entropy loss to evaluate the discrepancy between the predicted probability distribution of the model and the actual labels. We formulate the local loss function of client *i* as follows:

9 $${{\mathcal{L}}}_{i}^{cls}=-\frac{1}{| {{\mathcal{D}}}_{i}| }\mathop{\sum }\limits_{t=1}^{| {{\mathcal{D}}}_{i}| }\mathop{\sum }\limits_{c=1}^{C}{{\mathcal{Y}}}_{tc}\log (\widehat{{{\mathcal{Y}}}_{tc}})$$

where ${{\mathcal{D}}}_{i}$ is the local dataset of client *i* with size ${{\mathcal{D}}}_{i}$, *C* is the number of classes, $\widehat{{{\mathcal{Y}}}_{tc}}$ is the predicted probability that ${{\mathcal{X}}}_{t}$ belongs to class *c*, and ${{\mathcal{Y}}}_{tc}$ is the ground truth label. In detail, $\widehat{{{\mathcal{Y}}}_{t}}=[\widehat{{{\mathcal{Y}}}_{t1}},\widehat{{{\mathcal{Y}}}_{t2}},\ldots ,\widehat{{{\mathcal{Y}}}_{tC}}]$ is the one-hot coding output of the prompted foundation model, $\widehat{{{\mathcal{Y}}}_{t}}={\mathcal{F}}({{\mathcal{X}}}_{t},{\mathcal{P}})$.

For the segmentation task, we employ two loss functions to supervise segmentation learning. First, in image segmentation tasks, each pixel is typically labeled, indicating whether it belongs to the foreground or background. In this context, the entire image can be treated as a series of independent binary classification problems, applying BCE (Binary Cross-Entropy) loss to each pixel. The final loss value is the average of the losses across all pixels. Additionally, segmentation performance can be evaluated by assessing the similarity between two sets (the predicted segmentation ${\mathcal{S}}$ and the ground truth mask ${\mathcal{T}}$). In our paper, we leverage soft dice loss for evaluation, which implies that the model’s output doesn’t need to be strictly converted into binary labels. The overall segmentation loss function of client *i* is defined as follows:

10 $$\begin{array}{l}{{\mathcal{L}}}_{BCE}=-\frac{1}{| {{\mathcal{D}}}_{i}| }\mathop{\sum }\limits_{t=1}^{| {{\mathcal{D}}}_{i}| }\mathop{\sum }\limits_{j=1}^{| {{\mathcal{S}}}_{t}| }({{\mathcal{Y}}}_{j}\log (\widehat{{{\mathcal{Y}}}_{j}})+(1-{{\mathcal{Y}}}_{j})\log (1-\widehat{{{\mathcal{Y}}}_{j}})\\ {{\mathcal{L}}}_{Dice}=-\frac{1}{| {{\mathcal{D}}}_{i}| }\underset{t=1}{\overset{| {{\mathcal{D}}}_{i}| }{\sum \left(1-\frac{2{\sum }_{j=1}^{| {{\mathcal{S}}}_{t}| }\widehat{{{\mathcal{Y}}}_{j}}{{\mathcal{Y}}}_{j}}{{\sum }_{j=1}^{| {{\mathcal{S}}}_{t}| }{\widehat{{{\mathcal{Y}}}_{j}}}^{2}+{\sum }_{j=1}^{| {{\mathcal{S}}}_{t}| }{{{\mathcal{Y}}}_{j}}^{2}}\right)}}\\ {{\mathcal{L}}}_{i}^{seg}={{\mathcal{L}}}_{BCE}+{{\mathcal{L}}}_{Dice},\end{array}$$

where ${{\mathcal{S}}}_{t}$ is the predicted segmentation mask for input image ${{\mathcal{X}}}_{t}$ with totally $| {{\mathcal{S}}}_{t}|$ pixels, $\widehat{{{\mathcal{Y}}}_{j}}$ represents the model’s estimated probability that the given pixel *j* belongs to the positive class. Similarly, ${{\mathcal{T}}}_{t}$ is the ground truth mask for ${{\mathcal{X}}}_{t}$, and ${{\mathcal{Y}}}_{j}$ is the corresponding true label of the *j*th pixel. Specifically, ${{\mathcal{S}}}_{t}$ is obtained by prompted foundation model as ${{\mathcal{S}}}_{t}={\mathcal{F}}({{\mathcal{X}}}_{t},{\mathcal{P}})$, while $\widehat{{{\mathcal{Y}}}_{j}}\in {{\mathcal{S}}}_{t}$.

### Evaluation metrics

#### Image classification

We employ two metrics to evaluate the performance of classification tasks. Firstly, we use accuracy as the primary metric for evaluating multi-classification tasks, which measures the proportion of correctly classified instances among the total instances. Secondly, we employ the AUC (Area Under the Curve) metric to evaluate binary classification tasks on imbalanced datasets in melanoma diagnosis. Accuracy represents the proportion of correctly classified samples out of the total number of samples. The ROC curve is a graphical representation used to visually assess the performance of a classifier across all possible classification thresholds. The x-axis of the ROC curve represents the False Positive Rate (FPR), while the y-axis represents the True Positive Rate (TPR). The Area Under the Curve (AUC) measures the area under the ROC curve and reflects the classifier’s ability to distinguish between positive and negative cases.

#### Image segmentation

We adopt three metrics to evaluate the segmentation performance, Dice Similarity Coefficient (DSC), Intersection over Union (IoU), and the 95th percentile Hausdorff distance (HD95). DSC is a metric commonly used to evaluate the similarity between the predicted segmentation ${\mathcal{S}}$ and the ground truth segmentation ${\mathcal{T}}$ in image analysis tasks. It measures how closely the predicted segmentation matches the ground truth by considering both the precision and recall of the segmentation. It’s formulated as follows:

11 $$DSC({\mathcal{S}},{\mathcal{T}})=\frac{2| {\mathcal{S}}\cap {\mathcal{T}}| }{| {\mathcal{S}}| +| {\mathcal{T}}| }.$$

IoU measures the overlap between the predicted segmentation result ${\mathcal{S}}$ and the ground truth mask ${\mathcal{T}}$. IoU assesses the accuracy of segmentation by calculating the ratio of the intersection area to the union area of the predicted region and the actual region. IoU is defined as follows:

12 $$IoU({\mathcal{S}},{\mathcal{T}})=\frac{| {\mathcal{S}}\cap {\mathcal{T}}| }{| {\mathcal{S}}\cup {\mathcal{T}}| }.$$

HD95 is a modified version of the Hausdorff distance, providing a more robust assessment of the worst-case errors in segmentation results. The Hausdorff distance is a metric that measures the maximum discrepancy between predicted segmentation boundaries and ground truth boundaries, which is defined as:

13 $$HD({\mathcal{S}},{\mathcal{T}})=max\left\{\mathop{\sup }\limits_{u\in {{\mathcal{B}}}_{{\mathcal{S}}}}\mathop{\inf }\limits_{v\in {{\mathcal{B}}}_{{\mathcal{T}}}}d(u,v),\mathop{\sup }\limits_{v\in {{\mathcal{B}}}_{{\mathcal{T}}}}\mathop{\inf }\limits_{u\in {{\mathcal{B}}}_{{\mathcal{S}}}}d(u,v)\right\},$$

where ${{\mathcal{B}}}_{{\mathcal{T}}}$ and ${{\mathcal{B}}}_{{\mathcal{S}}}$ are the bound of masks ${\mathcal{T}}$ and ${\mathcal{S}}$, sup and inf are the supremum and infimum, *d*(*u*, *v*) represents the Euclidean distance between the points *u* and *v*. Unlike the standard Hausdorff distance, HD95 does not consider the maximum distance. Instead, it takes the 95th percentile of all point-to-point distances:

14 $$HD95({\mathcal{S}},{\mathcal{T}})=max\left\{{\mathop{\sup }^{95}_{u\in {{\mathcal{B}}}_{{\mathcal{S}}}}\limits}\,\mathop{\inf }\limits_{v\in {{\mathcal{B}}}_{{\mathcal{T}}}}d(u,v),{\mathop{\sup }^{95}_{v\in {{\mathcal{B}}}_{{\mathcal{T}}}}\limits}\,\,\mathop{\inf }\limits_{u\in {{\mathcal{B}}}_{{S}}}d(u,v)\right\},$$

where $\mathop{\sup }\limits^{95}$ is the 95th percentile maximum value.

## Supplementary information

Supplementary Information

## Data Availability

The datasets used in this paper are publicly available. The diabetic retinopathy classification datasets are from four datasets, which are available at https://www.kaggle.com/competitions/aptos2019-blindness-detection/data, https://www.kaggle.com/competitions/diabetic-retinopathy-detection/data, https://www.adcis.net/en/third-party/messidor/, and https://www.adcis.net/en/third-party/messidor2/, respectively. The melanoma classification dataset is available at https://challenge.isic-archive.com/data/#2019. The polyp segmentation datasets are from five datasets, which are available at https://aistudio.baidu.com/datasetdetail/233488/0. The prostate segmentation dataset is available at https://liuquande.github.io/SAML/.

## Code availability

To facilitate reproducibility, the source code is available at https://github.com/cyldg/Fed-GPL.
