# Supplementary material for: Federated generative prompt learning with vision foundation models: universal efficient multi-center medical image analysis
Source: NPJ Digit Med. 2026 Jun 10;9:713. doi: 10.1038/s41746-026-02866-1 (PMC13594208; doi:10.1038/s41746-026-02866-1)
Supplement: Supplementary file 1 [file 41746_2026_2866_MOESM1_ESM.pdf]

Supplementary Materials

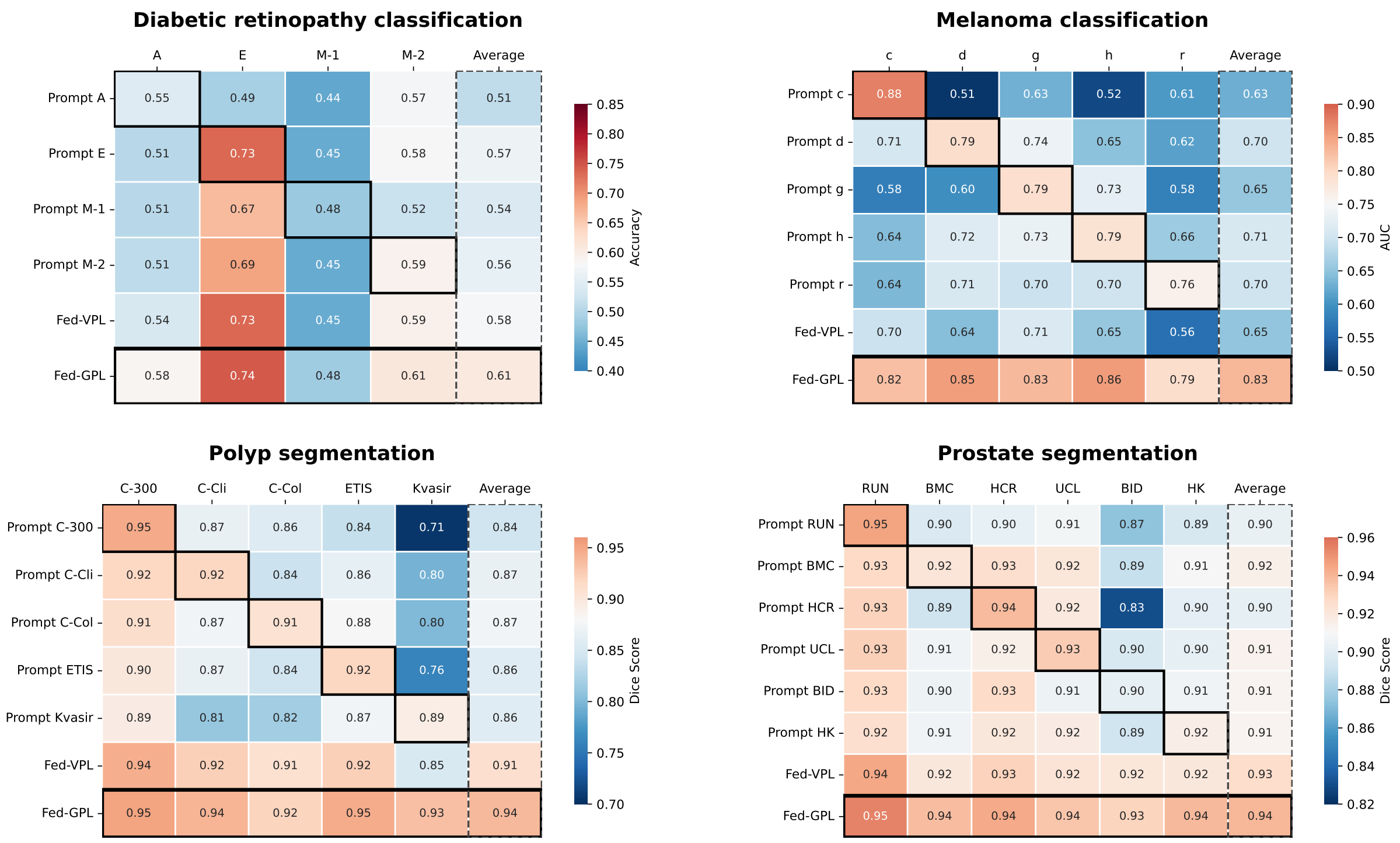

Figure S1: Cross-domain performance heatmaps across four medical imaging tasks. Each subplot presents the zero-shot generalization of various prompts and the proposed federated methods: (a) Diabetic retinopathy classification (Accuracy), (b) Melanoma classification (AUC), (c) Polyp segmentation (Dice Score), and (d) Prostate segmentation (Dice Score). The color scale represents performance relative to the task average: deeper red indicates higher performance, while deeper blue signifies lower performance. Black solid boxes on the diagonal highlight source-domain results, and the bottom-most row (solid box) identifies our Fed-GPL method, demonstrating superior generalization. The rightmost column (dashed box) reports the average performance across all test domains.

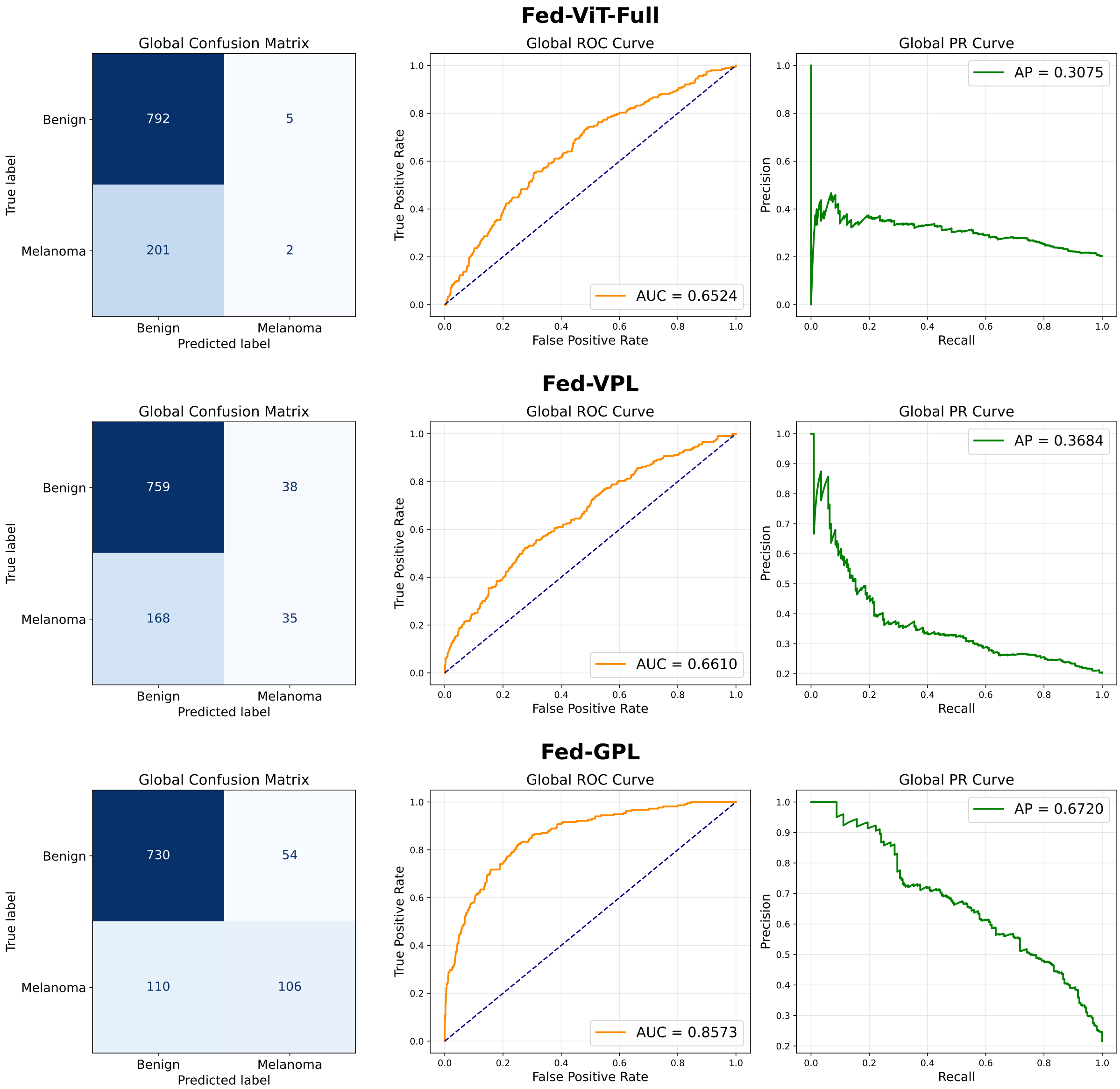

Figure S2: Confusion matrices, ROC curves, and precision–recall (PR) curves on melanoma classification. We provide global confusion matrices (left), ROC curves (middle), and PR curves (right) to complement the main quantitative results. All methods are evaluated under identical experimental settings; rows from top to bottom correspond to Fed-ViT-Full, Fed-VPL, and Fed-GPL. Compared with Fed-ViT-Full and Fed-VPL, Fed-GPL consistently yields better class separation and a more favorable precision–recall trade-off, which is particularly important for challenging cases and the minority class (melanoma).

Table S1: Performance evaluation on the Melanoma classification task for cross-center generalization using AUC. The table presents the AUC values across different medical centers as well as the average performance. The best results are highlighted in bold. Fed-GPL consistently achieves the highest AUC values across all medical centers, demonstrating its superior cross-center generalization ability.

method	Melanoma (AUC) ↑					
	c	*d*	*g*	*h*	*r*	*Avg*
Fed-ResNet	48.52	38.93	47.28	44.12	30.23	42.82
Fed-ViT	63.47	56.05	30.77	51.59	43.54	49.09
Fed-ViT-Full	66.65	56.24	54.22	62.33	44.35	56.76
Fed-VPL (ours)	63.82	49.32	66.13	47.41	59.19	57.17
Fed-GPL (ours)	**74.97**	**84.92**	**81.59**	**73.25**	**67.45**	**76.44**

Table S2: Performance evaluation on the Polyp segmentation task for cross-center generalization, reported using DSC and HD95. The best DSC performance is highlighted in bold, and the best HD95 performance is underlined. The table compares different methods across five polyp datasets along with the average performance. Fed-GPL outperforms the other methods in both DSC and HD95, achieving the best overall results and demonstrating its robust cross-center generalization abilit

Method	Metric	Polyp					
		C-300	*C-Cli*	*C-Col*	*ETIS*	*Kvasir*	*Avg*
Fed-U-Net	DSC↑	89.34	88.95	82.46	84.20	80.34	85.06
	HD95↓	12.53	23.45	40.94	38.62	32.84	29.78
Fed-DeepLabV3	DSC↑	82.54	85.23	77.04	70.49	88.30	80.72
	HD95↓	60.38	22.54	42.67	295.66	<u>13.87</u>	87.02
SAM	DSC↑	90.09	82.69	81.97	57.34	78.87	78.19
	HD95↓	<u>6.23</u>	12.81	16.92	291.40	34.66	72.40
Fed-SAM	DSC↑	92.38	89.95	90.33	88.54	80.69	88.38
	HD95↓	6.85	18.66	<u>13.57</u>	<u>26.84</u>	50.57	23.30
Fed-VPL (Ours)	DSC↑	92.57	92.66	89.23	90.76	82.56	89.56
	HD95↓	7.45	15.49	21.34	33.69	42.45	24.10
Fed-GPL (Ours)	DSC↑	**94.65**	**92.78**	**90.32**	**91.96**	**90.54**	**92.05**
	HD95↓	6.53	<u>11.45</u>	15.45	30.47	39.28	<u>20.64</u>

Table S3: Distribution of diabetic retinopathy (DR) grades across domains.

DR Grade	*Aptos*	*EyePACS*	*Messidor-1*	*Messidor-2*	Total
No DR	1805	25810	546	1017	29178
Mild DR	370	2443	153	270	3236
Moderate DR	999	5292	247	347	6885
Severe DR	193	873	254	75	1395
Proliferative DR	295	708	-	35	1038
Total	3662	35126	1200	1744	41732

Table S4: Distribution of skin lesion classes across different artifact domains.

Class	*Clean*	*Dark corner*	*Gel bubble*	*Ruler*	*Hair*	Total
Benign	3951	3477	2713	884	7868	18893
Melanoma	781	1132	837	274	1102	4126
Total	4732	4609	3550	1158	8970	23019

Table S5: Performance evaluation on the binary melanoma classification task. Each column corresponds to one repeated random split. Each entry reports the AUC (%) averaged over all domains under the corresponding split. The last column reports the average performance across all splits. Best results are highlighted in bold.

Method	Split 1	Split 2	Split 3	Split 4	Split 5	Avg
Fed-ResNet	49.53	55.30	50.23	44.62	48.29	49.59 ± 3.85
Fed-ViT	62.89	63.59	58.67	61.45	62.12	61.74 ± 1.90
Fed-ViT-Full	63.78	62.53	61.90	63.12	62.85	62.84 ± 0.70
Fed-VPL (ours)	65.31	66.84	66.12	63.78	68.35	66.08 ± 1.70
Fed-GPL (ours)	**83.16**	**84.53**	**82.05**	**81.28**	**82.49**	**82.70 ± 1.23**

Table S6: Performance evaluation on the polyp segmentation task. Each column corresponds to one repeated random split. Each entry reports the DSC (%) averaged over all domains under the corresponding split. The last column reports the average performance across all splits. Best results are highlighted in bold.

Method	Split 1	Split 2	Split 3	Split 4	Split 5	Avg
Fed-U-Net	88.52	84.12	90.45	85.33	89.12	87.51 ± 2.65
Fed-DeepLabV3	83.77	88.54	85.12	89.45	86.32	86.64 ± 2.34
SAM	78.19	85.42	80.15	86.78	79.94	82.10 ± 3.79
Fed-SAM	90.76	91.45	89.32	90.15	91.04	90.54 ± 0.83
Fed-VPL (Ours)	90.96	91.88	90.12	91.45	90.67	91.02 ± 0.69
Fed-GPL (Ours)	**93.46**	**93.88**	**92.15**	**94.12**	**93.57**	**93.44 ± 0.77**

Table S7: Performance comparison on diabetic retinopathy classification using imbalance-aware metrics. Results are averaged over repeated random splits. Macro-F1 reports both the overall macro-averaged score and per-class F1 scores. Balanced Accuracy (BalAcc) and MCC are reported as overall metrics. Class definitions are as follows: Class 1—No DR, Class 2—Mild DR, Class 3—Moderate DR, Class 4—Severe DR, and Class 5—Proliferative DR.

Method	Macro-F1	F1-score					BalAcc	MCC
		Class 1	Class 2	Class 3	Class 4	Class 5		
Fed-ViT-Full	0.1618	0.7142	0.0206	0.0741	0	0	0.2015	0.0081
Fed-VPL	0.1998	0.7200	0.0625	0.2167	0	0	0.2234	0.0950
Fed-GPL	0.3317	0.7532	0.1835	0.3354	0.2142	0.1739	0.4139	0.3241

Table S8: Performance comparison on melanoma classification using imbalance-aware metrics. Results are averaged over repeated random splits. Macro-F1 reports both the overall macro-averaged score and per-class F1 scores. Balanced Accuracy (BalAcc) and MCC are reported as overall metrics. Class definitions are as follows: Class 1—Benign, Class 2—Melanoma.

Method	Macro-F1	F1-score		BalAcc	MCC
		Class 1	Class 2		
Fed-ViT-Full	0.4519	0.8849	0.0190	0.5018	0.0173
Fed-VPL	0.5562	0.8811	0.2313	0.5550	0.1796
Fed-GPL	0.7314	0.8990	0.5638	0.7109	0.4735

Table S9: Per-task trainable parameter composition per client.

Task	Backbone	$\lvert\theta_G\rvert$	$\lvert\theta_P\rvert$	$\lvert\theta_H\rvert$	$\lvert\theta_{MD}\rvert$	Sum
DR	ViT-B	7087301	7680	3845	–	7098826
Melanoma	ViT-B	29529797	36864	1538	–	29568199
Polyp	SAM ViT-B	15617600	1179648	–	4058340	20855588
Prostate	SAM ViT-B	2033984	49152	–	4058340	6141476

Tables S10: Hyperparameter sensitivity and ablation studies across four tasks. We evaluate the effects of learning rate, the number of local training epochs, prompt initialization strategy, and the number of generator heads, while keeping all other hyperparameters unchanged. Metrics are ACC for DR, AUC for Melanoma, and Dice for Polyp/Prostate. Best results for each task within each block are shown in **bold**.

Setting	DR (ACC↑)	Melanoma (AUC↑)	Polyp (Dice↑)	Prostate (Dice↑)
**Learning rate ablation**				
1×10^{-6}	**60.63**	**83.16**	91.47	92.89
5×10^{-6}	59.36	80.53	90.52	92.56
1×10^{-5}	58.49	81.39	91.29	92.86
5×10^{-5}	58.25	79.41	93.17	93.08
1×10^{-4}	57.81	76.83	**93.86**	**93.97**
**Local training epochs ablation**				
1	56.75	72.81	91.32	90.42
2	**60.63**	83.16	**93.86**	**93.97**
3	60.57	**84.06**	92.95	93.67
4	59.87	82.29	93.08	93.44
**Prompt initialization ablation**				
Zero start	**60.63**	**83.16**	**93.86**	**93.97**
Random	58.18	82.06	93.17	93.56
**Number of generator heads ablation**				
6	59.30	81.34	93.45	92.52
12	**60.63**	**83.16**	93.86	93.97
18	58.92	82.49	**94.06**	**94.25**
